# Understanding pellet population heterogeneity of *Aspergillus niger* in stirred tank and rocking motion bioreactors

**DOI:** 10.1007/s00253-026-13822-0

**Published:** 2026-05-08

**Authors:** Karin Engelbert, Tolue Kheirkhah, Charlotte Deffur, Fangxing Zhang, Henri Winter, Timothy Cairns, Sascha Jung, Heiko Briesen, Peter Neubauer, Stefan Junne, Vera Meyer

**Affiliations:** 1 Institute of Biotechnology, Chair of Applied and Molecular Microbiology, Technische Universität Berlin, Gustav-Meyer Allee 25, 13355 Berlin, Germany; 2 Institute of Biotechnology, Chair of Bioprocess Engineering, Technische Universität Berlin, Ackerstraße 76 ACK24, 13355 Berlin, Germany; 3 School of Life Sciences Weihenstephan, Chair of Process Systems Engineering, Technical University of Munich, Gregor-Mendel-Straße 4, 85354 Freising, Germany; 4 Department of Chemistry and Bioscience, Aalborg University, Niels Bohrs Vej 8, 6700 Esbjerg, Denmark

**Keywords:** Morphology engineering, Macromorphology, Micromorphology, Talcum microparticles, Seed culture, Culture history, Shear force

## Abstract

**Abstract:**

The filamentous fungus *Aspergillus niger* is a well-established cell factory in biotechnology. Its productivity depends on macromorphological development which remains difficult to control, partly because the relationship between seed culture and reactor-specific shear force conditions has not been systematically investigated. This study examined how high or low shear forces affect pellet development at both micro- and macromorphological levels in stirred-tank reactors (STR, high shear regime) and rocking-motion bioreactors (RMB, low shear regime). *A. niger* seed cultures with initially either large or small pellets were used to inoculate batch STR or RMB. Comparable cultivation conditions were applied so that fermentations differed mainly in shear force regime. Growth characteristics and pellet macromorphologies were analysed using 2D and 3D image analyses, enabling us to classify pellets according to three different classes based on their inner pellet architecture. The distribution of these classes depended on both the macromorphologies of the seed culture and the reactor type. Under high shear forces in the STR, pellets underwent breakage shortly after stirrer activation, were limited in their size to an average diameter of 500–600 µm, and formed a homogeneous population. In addition, broken pellets occurred predominantly under STR conditions. In contrast, cultivations in RMB preserved the initial pellet architecture, allowed the formation of larger pellets (median diameter ~ 800 µm) and supported pellet fusion, thus resulting in a more heterogeneous macromorphological population. Notably, glucose uptake rate correlated with the surface-to-volume ratio of the pellet populations, i.e., glucose became faster consumed under STR conditions accompanied with lower biomass yields and higher protein secretion. Citric acid production, however, was detectable in both STR and RMB only when reactors were inoculated with seed cultures characterised by a loose pellet morphology. Overall, our study demonstrates how shear regime and seed culture morphology jointly shape pellet architecture, population heterogeneity and productivity in scale-up processes. Such a comprehensive understanding of morphological developments is instrumental for optimising bioprocesses and future predictive modelling approaches.

**Key points:**

• *2D/3D analysis of defined seed cultures in different shear-induced environments*

• *High shear restricts and homogenises pellets, while low shear maintains heterogeneity*

• *Highest citric acid and total protein levels were found in smaller, compact pellets. *

**Supplementary Information:**

The online version contains supplementary material available at 10.1007/s00253-026-13822-0.

## Introduction

Submerged cultivation of *Aspergillus niger* is widely employed in biotechnological applications, ranging from waste treatment to production of pharmaceutical compounds, enzymes and organic acids (Meyer et al. 2020; El Enshasy 2022). Despite its extensive use, controlling *A. niger* macromorphology is a significant challenge for ensuring consistent productivity across multiple product classes (Cairns et al. 2021; El Enshasy 2022). *A. niger* can develop three distinct forms in submerged culture: (i) roughly spherical pellets which can grow up to several millimetres in diameter; (ii) fully dispersed mycelia and (iii) clumps, which are intermediates characterised by loose hyphal entanglement (Cairns et al. 2022; Meyer et al. 2021; Müller et al. 2022). These visible morphological forms are referred to as macromorphology, observable at the pellet or culture level. However, when examining filamentous development at the hyphal level, the term micromorphology is employed, emphasising finer structural details such as total hyphal length and the number of hyphal tips (Dinius et al. 2023). The formation of diverse macromorphologies in *A. niger* is hypothesised to be controlled at the microscale by spore agglomeration, and subsequent apical hyphal growth and branching (Cairns et al. 2022; Engelbert et al. 2025). This is coupled with probable interaction of larger fungal entities with one another and the bioreactor system on a macroscale (Böl et al. 2021). Such interplays can lead to morphologically heterogeneous populations, which might be advantageous for the adaptation of the population in the natural environment, but are often seen as disadvantageous in industrial bioprocesses (Zacchetti et al. 2018). Therefore, investigating *A. niger* morphology at both micro- and macro-levels will improve any holistic understanding crucial for optimising process performance (Müller et al. 2023).

*A. niger* macromorphology affects broth rheology, thereby influencing the power input and, subsequently, the volumetric oxygen transfer and nutrient mixing within the submerged culture (Veiter et al. 2018). While dispersed growth leads to non-Newtonian characteristics posing challenges in mixing and mass transfer, pelleted cultures exhibit a more Newtonian fluid behaviour (Wucherpfennig et al. 2013). On the other hand, oxygen and nutrient diffusion within pellets can be limited depending on their diameter and compactness (Schmideder et al. 2019b; Deffur et al. 2024). For pellets with varied hyphal density in the outer pellet region, an oxygen penetration depth of 90–290 µm was recently measured using microelectrode and X-ray microtomography, proving this outer region to be metabolically active (Deffur et al. 2024). Therefore, to reduce mass transfer limitation within pellets, small pellets are generally the preferred morphology, particularly at larger scales (Lin et al. 2010). The macromorphology of *A. niger* is ultimately believed to play a key role in growth which in turn can impact metabolite production and cells’ productivity (Cairns et al. 2019b; Dinius et al. 2024). Several factors are reported to influence macromorphology including the spore concentration (Li et al. 2022; Engelbert et al. 2025), nitrogen source (Ikram-Ul-Haq et al. 2005; Li et al. 2022), pH (Colin et al. 2013; Buffo et al. 2021), osmolality (Dittmann et al. 2019; Tesche et al. 2019), oxygen availability (Fazenda et al. 2010) and the shear force regime (Kelly et al. 2004; Böl et al. 2021). Nevertheless, achieving optimal conditions for the controlled development of certain morphologies is challenging, particularly when transferring cultures across scales (Meyer et al. 2021). Recently, the application of microparticles, in general known as microparticle enhanced cultivation (Laible et al. 2021), has emerged as a reliable method in changing macromorphology in pellet-forming fungi with coagulative spores (Walisko et al. 2012; Böl et al. 2021; Engelbert et al. 2025). It was shown that the addition of talcum to a maximum concentration of 10 g L^−1^ is effective in controlling macromorphology and, notably, pellet diameter in *A. niger* under a low shear force environment (Kurt et al. 2018; Kheirkhah et al. 2023). This was possible under controlled cultivation conditions in a 2-dimensional rocking-motion bioreactor (RMB), where mixing takes place without stirrers. Such bioreactors are generally used in seed trains up to production scale in single-use bioprocesses for both microbial and cell culture applications (Junne and Neubauer 2018).

The shear force regime is recognised as one of the crucial factors impacting the macromorphology of *A. niger* (Kunz and King 2022). In stirred-tank reactors (STR), commonly used as production systems for *Aspergillus* spp*.*, high agitation rates are required to ensure a sufficient gas-mass transfer and mixing. Consequently, the combination of vigorous agitation and aeration exposes the mycelium to considerably high shear forces potentially causing hyphal fragmentation and pellet breakage. The disruption of single hyphae can shape the morphology on a microscale where intense fragmentation can also affect the macroscale, e.g., by reducing the integrity of the pellet (Kelly et al. 2006). In turn, clumping and pellet breakage influence the macromorphology directly (Müller et al. 2022). In contrast to a STR, a RMB maintains shear forces comparable to common shake flask cultivations (Kheirkhah et al. 2023). A 2-dimensional rocking-motion bioreactor with vertical and horizontal movement has been shown to provide sufficiently high oxygen gas-mass transfer rates while maintaining a low shear force regime. In this system, power input depends on both rocking angle and speed, but it is estimated to be approximately 35 W m^−3^ (for water under typical operating conditions) (Seidel et al. 2022), which corresponds to roughly 10–20% compared to the power input in the STR (Montes-Serrano et al. 2023). Despite the lower shear force regime, the RMB can achieve a volumetric gas-mass transfer coefficient (k_L_a) for oxygen of up to 600 h^−1^ (Junne et al. 2013; Seidel et al. 2022). Reaching such a k_L_a value in a lab-scale STR under lower shear force regime is not feasible, as the required stirring rate (power input) will be insufficient (Kheirkhah et al. 2023).

Assessing macromorphology under various cultivation conditions has long relied on several established analytical tools (Krull et al. 2013). Typically, images of the population are examined using semi-automated analyses, allowing the quantification of some macromorphological structures such as pellet diameter (Cairns et al. 2019a; Müller et al. 2022). Recently, analysis of micromorphology through innovative techniques such as X-ray-based microcomputed tomography (µ-CT) at laboratory scale (Schmideder et al. 2019a) or in high-throughput approaches via synchrotron radiation-based microcomputed tomography (SR-µ-CT) has been proposed (Müller et al. 2023). These techniques have enabled the analysis of the 3D micromorphological structure of fungal pellets, offering a completely new perspective on inner pellet architecture and the influence of external parameters on process performance. Using SR-µ-CT, we recently introduced, for the first time, a classification system for fungal pellets based on their inner architectures, defined as the three-dimensional spatial organisation and distribution of the hyphal network within a pellet (Engelbert et al. 2025). In brief this demonstrated: the agglomeration of spores at an early growth phase leads to the formation of pellets with one centred spore agglomerate growing uniformly outwards (class I), while pellets of class II are formed during a later growth phase by the attachment of multiple spore agglomerates. Pellets of class III, on the other hand, originate from mature pellets and pellet fragments, by loosely entangled hyphae and often have highly irregular non-spherical shapes.

This study has two main objectives; firstly, to quantify how the average pellet diameter present in *A. niger* seed culture impacts morphological development during subsequent bioreactor cultivation. Secondly, to determine how morphology differs between high or low shear force cultivation regimes. Therefore, seed cultures with distinct pellet populations were obtained from shake flask cultures supplemented with either 1 or 10 g L^−1^ talcum. Bioreactors were operated under otherwise similar cultivation conditions, with the key distinction being the higher mechanically induced shear forces in the STR compared to the RMB. The resulting pellets were subsequently analysed using both 2D and 3D image analyses down to the hyphae scale. Finally, the implications of the morphological differences on overall process performance were assessed, including growth parameters and two product groups, mainly used in industrial biotechnology, namely secreted proteins and citric acid. To the best of our knowledge, this study is the first to combine controlled seed culture heterogeneity with quantitative 2D and 3D morphological analysis to systematically compare the effects of reactor-specific shear environments on fungal pellets.

## Materials and methods

### Strain and spore solution preparation

Recombinant *A. niger* strain ÖV4.10, constructed by Richter et al. (2014), was used in this study (available from the corresponding author upon request). Solid agar was inoculated with spores and incubated for 5 days at 30 °C on solid complete medium (CM) (Meyer et al. 2010). Harvesting and preparation of fresh spore solutions were carried out on the day of cultivation as described in Kheirkhah et al. (2023).

### Seed culture inoculum preparation

For preparation of the inoculum, *A. niger* spores (5 × 10^6^ spores mL^−1^) were grown for 12 or 24 h in unbaffled flasks (250 mL) supplemented with either 1 g L^−1^ talcum or 10 g L^−1^ talcum at 150 rpm (Engelbert et al. 2025). Biomass was harvested during the exponential growth phase (12 h), under sterile conditions by using small-pored pleated filters and washing with minimal medium (MM), which is routinely used as a standard salt medium for *A. niger* bioreactor cultivations. MM contained the following (per litre): 4.5 g of NH_4_Cl, 1.5 g of KH_2_PO_4_, 0.5 g of KCl, 0.5 g of MgSO_4_ × 7 H_2_O, 0.1% (v v^−1^) modified Vishniac trace element stock solution, and 8 g glucose (Jørgensen et al. 2010). Wet biomass was weighed and divided into two equal parts under sterile conditions. Each part containing 200 g wet weight (0.4% w w^−1^ inoculation ratio) was transferred into a bioreactor inoculation flask together with a defined liquid volume (750 mL) of MM.

### Bioreactor cultivation

Submerged cultivations were performed in a 7.5 L BioFlo310 bioreactor (New Brunswick Scientific, NJ, USA) and 20 L 2-dimensional rocking-motion bioreactor CELL-tainer® CT20 (Celltainer Biotech, Winterswijk, The Netherlands). Detailed descriptions of the cultivation settings were given previously (Jørgensen et al. 2010; Kurt et al. 2018; Kheirkhah et al. 2023). In brief, both reactors were operated with 5 L of MM supplemented with glucose at an initial concentration of 8 g L^−1^. All the components were sterilised by autoclaving at 121 °C for at least 20 min. The pH value of the media was adjusted to 3.0 with 1 M HCl prior to the inoculation and kept constant during the cultivation with automated 2 M NaOH addition. The bioreactors were inoculated simultaneously with the freshly harvested seed culture from the inoculation flasks. All connecting tubes had a sufficiently large opening of 4 mm to avoid pellet deformation. Both bioreactors operated at a temperature of 30 °C and aeration rate of 5 L min^−1^ (1 vvm). In the case of RMB, the rocker speed was controlled under DO-rpm mode, in which the dissolved oxygen level was maintained above 40% of saturation (see Supplementary Material 1) by adjusting the rocker speed (minimum speed of 15 and the maximum of 35 rpm). In the STR, stirrer speed was set to a constant 750 rpm immediately after the inoculation. Samples were taken every two hours for each reactor. In the STR, one extra sample was also taken for morphological analysis shortly after the stirrer activation. The bioreactor cultivations were carried out each in two biological replicates, thereafter denoted A and B.

### Analytical methods

#### Cell dry weight determination

A volume of 15 mL of culture aliquot was filtered through pre-weighed Whatman® filter paper (no. 41, WHA1441047, Whatman PLC, Keene, NH) using a vacuum pump. The filtrate was separated for metabolite analysis. The pellets were then washed with demineralised water in the filtration unit. For measurement of the cell dry weight (CDW), filter papers containing the pellets after filtration were dried until constant weight. The method was previously described by Fiedler et al. (2018) and Kheirkhah et al. (2023) for STR and RMB cultivations, respectively. CDW was determined in triplicates per sample. The reported CDW includes the combined biomass and residual talcum within the pellets, as talcum particles cannot be quantitatively separated from the biomass. Based on the inoculum conditions and the relative increase in dry weight during cultivation, the contribution of talcum is expected to be negligible.

#### Glucose, citric acid and total protein measurements from supernatant

The concentration of glucose and citric acid was measured using an Agilent 1200 high-performance liquid chromatography (HPLC) system (Agilent Technologies, Waldbronn, Germany) with a refractive index detector and a HyperRez™ XP Carbohydrate H^+^ column (300 × 7.7 mm, 8 µm, Thermo Fisher Scientific, Schwerte, Germany) with 0.1 M H_2_SO_4_ as eluent at a flow rate of 0.6 mL min^−1^ and a temperature of 65 °C. Total protein was quantified using the Bradford assay based Protein Assay Dye Reagent Concentrate (Bio-Rad Laboratories, Hercules, CA, USA) in a minimum of technical duplicates. Bovine Serum Albumin was used as the standard protein.

#### Macromorphological characterisation

Seed culture pellet formation was followed in higher magnification by transferring 15 µL of culture broth to a microscopic slide and using a Leica DM 5000 CS (Leica Microsystems, Wetzlar, Germany) Differential Interference Contrast (DIC) microscope. All further images for analysing macromorphology (2D) were obtained with a Leica S8APO stereomicroscope connected to a Leica MC120 HD camera. Defined amounts of culture broth and physiological saline solution (0.89% (w v^−1^) NaCl) were pipetted into an empty petri dish as well as 2 µL of 50% (v v^−1^) Tween® 20 (Sigma-Aldrich/Merck, Darmstadt, Germany) to avoid further agglomeration of pellets. The solution was swirled gently and images were taken as described before (Müller et al. 2022). Pellets and spores from seed culture with 1 g L^−1^ talcum were detected in the images using methods described in (Müller et al. 2022). However, for pellets with 10 g L^−1^ talcum, accurate recognition of the spore agglomerates within pellets was challenging, and some pellet boundaries were misidentified based on local threshold segmentation. Therefore, these images were analysed using our adapted image processing method described earlier (Engelbert et al. 2025), which segments the pellets based on their morphology without the detection of spore agglomerates. Pellet diameter distributions of distinct timepoints were statistically compared using the Mann–Whitney *U* test. The dispersed mycelia were detected with an approach that we have described previously (Müller et al. 2022).

#### Micromorphological characterisation

For micromorphology analyses, pellets from replicates A were freeze-dried followed by the procedure described here (Schmideder et al. 2019a). The freeze-dried pellets were analysed using synchrotron radiation-based microcomputed tomography (SR-µ-CT) as described in our recent study (Müller et al. 2023).

## Results

### Generation of seed cultures

**Fig. 1:**
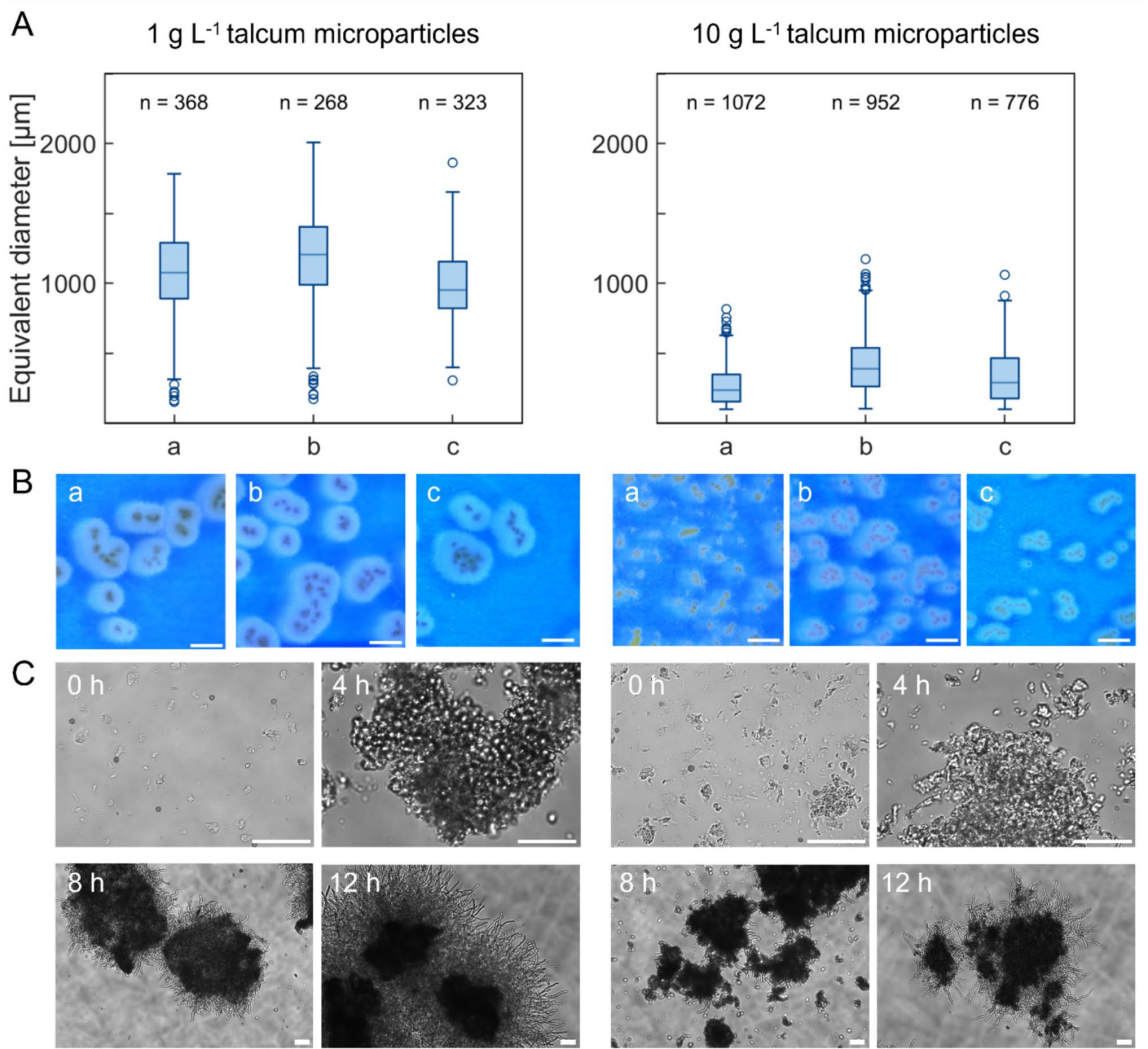
**A** Box plots representing the equivalent diameter of pellets for selected seed cultures cultivated with 1 g L^−1^ or 10 g L^−1^ talcum after 24 h (triplicates) with the number of analysed pellets (*n*). **B** Stereomicroscopic images of cultivations after 24 h illustrating morphological similarity between the triplicates. The scale bar represents 1000 µm. **C** Differential interference contrast (DIC) images highlighting steps in pellet formation. The scale bar represents 250 µm. Data originally published in Engelbert et al. (2025); adapted here

It was hypothesised that the seed culture morphology impacts subsequent bioreactor cultivation. Therefore, a preliminary screen in shake flasks was conducted to find optimal seed culture conditions. *A. niger* was grown under 48 distinct cultivation conditions in triplicate, and the size distribution of the pellets was quantified after 24 h using an adjusted automated image analysis workflow (Engelbert et al. 2025). A comprehensive discussion of those findings is provided in our recent publication (Engelbert et al. 2025). Of all the cultivation conditions, two were selected for seed culture generation based on various parameters (Fig. 1). Firstly, high homogeneity and reproducibility in pellet macromorphology must occur between technical replicates. Secondly, macromorphology must differ considerably between the respective cultivation conditions (i.e., > 400 µm difference in pellet diameter, Fig. 1).

The two selected populations differed by the amount of talcum microparticles added in the cultivation media (1 g L^−1^ or 10 g L^−1^, Fig. 1A and B), which is commonly used to adjust the pellet diameter (Wucherpfennig et al. 2012; Kheirkhah et al. 2023). Both conditions displayed a unimodal size distribution of the pellet diameter with at least 60% similarity among the populations of triplicate flasks, as determined by the overlap coefficient (Engelbert et al. 2025). In addition, the ratio of spores to talcum particles determined the number of spore-talcum agglomerates formed, representing the key difference between the two seed culture conditions. As depicted in Fig. 1C, this led to populations with different inner pellet architecture whereby talcum particles were integrated during the spore agglomeration phase (4 h). Additionally, the images show germinated spore agglomerates potentially fusing together (8 h) as well as mature pellets formed by several spore agglomerates (12 h). One reason to follow pellet formation was to ensure spores started to germinate and thereby entered the second agglomeration step as described by Grimm et al. (2004). This step is marked as a milestone in forming the basic architecture of pellets (Müller et al. 2022). Based on these observations along with cell dry weight (CDW) and substrate uptake to verify exponential growth, a cultivation time of 12 h was determined to be optimal for harvesting the seed cultures (Supplementary Fig. S1).

### Growth and pellet macromorphologies under high and low shear force regimes

**Fig. 2:**
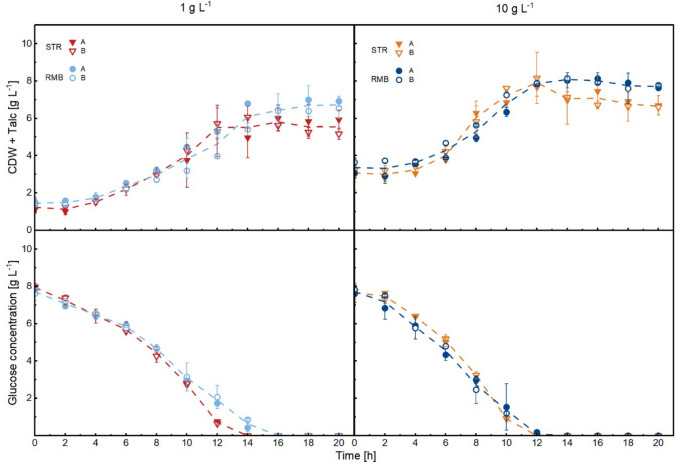
Cell dry weight and glucose concentration of identical *A. niger* pellet seed cultures in stirred-tank (triangles) and rocking-motion (circles) bioreactor cultivations. The pellet size of the seed cultures was adjusted either with 1 g L^−1^ or 10 g L^−1^ talcum microparticles. Runs were performed in biological duplicates: closed symbols represent replicate A, and open symbols represent replicate B. The mean value of both replicates is marked by the dashed line

**Fig. 3:**
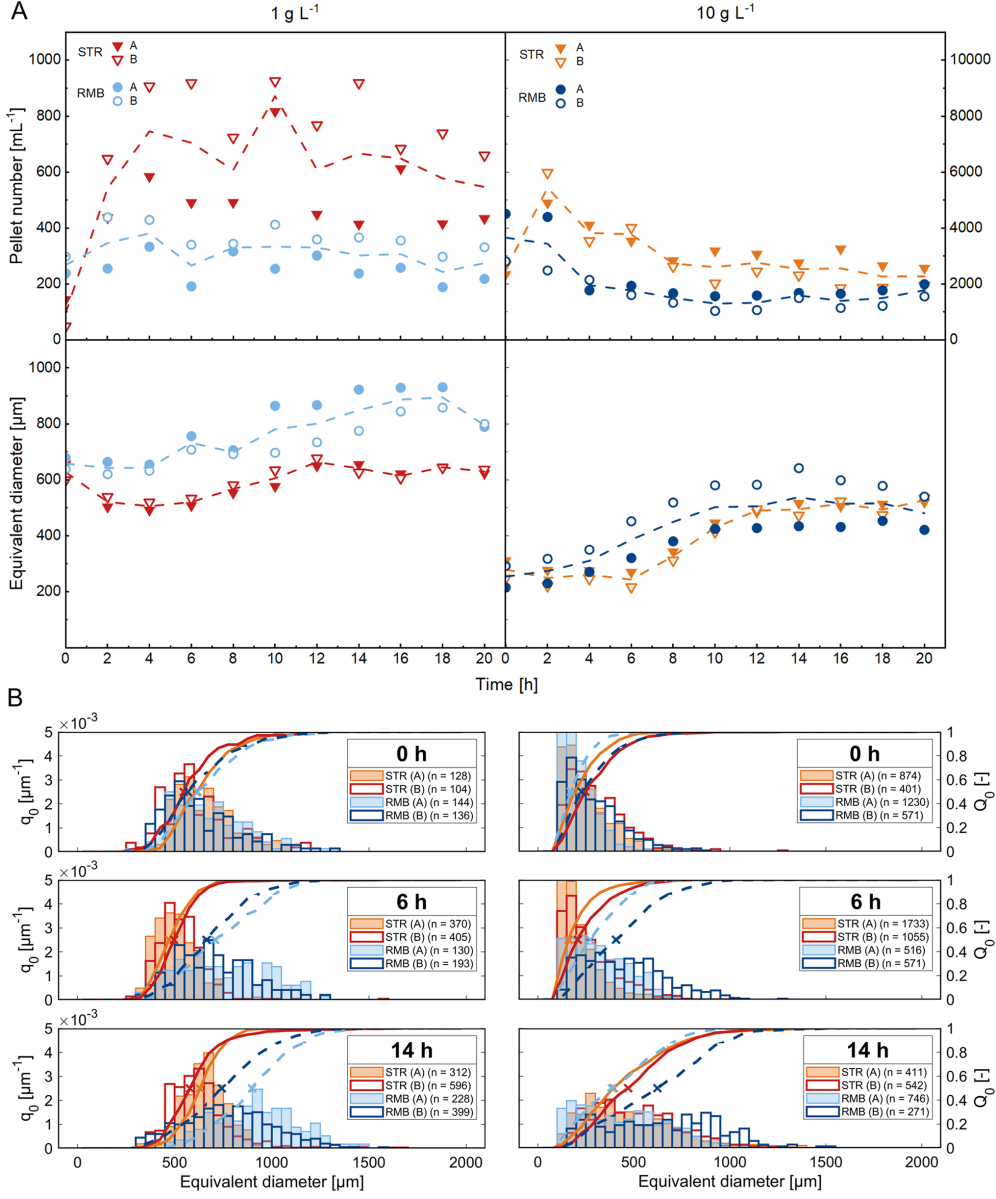
Development of pellet macromorphology, as obtained from 2D (area based) image analysis data. **A** Pellet number concentration and equivalent diameter of pellets for stirred-tank (STR, triangles) and rocking-motion (RMB, circles) bioreactor cultivations. The pellet number concentration was determined based on the number of counted pellets in a defined volume. Runs were performed in biological duplicates: closed symbols represent replicate A, and open symbols represent replicate B. For each time point, several hundred pellets were analysed, the overall population pellet diameter is represented by the median and the dashed line shows the mean value of both replicates. **B** Comparison of equivalent pellet diameter distributions. Histograms represent normalised frequency distributions (q₀) with a bin size of 50 µm, while the solid (STR) and dotted (RMB) lines show the corresponding cumulative distributions (Q₀). The “X” indicates the median diameter and (*n*) indicates the number of pellets analysed. Selected timepoints are shown; the full data set is provided in Supplementary Fig. S2a, b

To determine how the shear force regime in cultivations impacts growth behaviour and macromorphology, the STR and RMB were inoculated with the respective seed cultures (each bioreactor with 200 g of wet biomass). For simplicity, the cultivation conditions were labelled as follows: when 1 g L^−1^ of talcum was added to the seed culture, the STR and RMB cultivations were denoted STR1 and RMB1, respectively. Similarly, with 10 g L^−^^1^ of talcum, the cultivations were named STR10 and RMB10. The general cultivation behaviour was characterised by analysing CDW and glucose concentration (Fig. 2) over time as well as macromorphological development of the pellet diameter distributions (Supplementary Fig. S2a, b). Microscopy images from the cultivations in both bioreactors (STR vs. RMB) were analysed using 2D image analysis (see Materials and methods) and used to calculate the pellet number concentration (i.e., number of pellets per unit volume (mL^−1^)) and their area equivalent circular diameter (equivalent diameter, Fig. 3). Key parameters of growth and macromorphology are summarised in Table 1.

**Table 1:** Growth, productivity, macro- and micromorphological parameters of *A. niger* cultivations in stirred tank and rocking-motion bioreactor. Specific growth rates (µ) were calculated over the growth phase: 6–12 h for STR 1 g L^−1^, 6–14 h for RMB 1 g L^−^^1^ and 6–10 h for STR and RMB 10 g L^−^^1^ cultures. R_s_ and q_s_ are given as average values over the period of glucose uptake. Details of the calculations can be found in the Supplementary Materials. Comparative values for macro- and micromorphology are reported at 14 h, when glucose uptake had stopped and pellet architecture had essentially formed. The remaining parameters with stable trends were averaged over 20 h

		1 g L^−1^		10 g L^−1^	
		STR	RMB	STR	RMB
Growth	ΔCDW_max_* [g L^−1^]	4.80 ± 0.1	5.31 ± 0.2	4.84 ± 0.1	4.72 ± 0.2
	Specific growth rate µ [h^−1^]	0.15	0.12	0.15	0.12
	Substrate uptake rate R_s_ [g L^−1^ h^−1^]	0.60	0.51	0.84	0.64
	Specific substrate uptake rate q_s_ [g g ^1^ h^−1^]	0.13	0.10	0.17	0.14
	Biomass yield Y _x/s_ [g g^−1^]	0.58	0.68	0.63	0.62
Productivity	Extracellular protein [µg mL^−1^] at 20 h	35.73 ± 11.76	6.21 ± 6.21	36.15 ± 0.04	4.47 ± 4.47
	Citric acid [g L^−1^] at 20 h	2.32 ± 0.68	0	0.84 ± 0.38	0
Macromorphology	Pellet number concentration [mL^−1^] at 14 h	666 ± 253	302 ± 65	2533 ± 221	1588 ± 90
	Inter quartile range [µm] at 14 h	153 ± 9	308 ± 11	337 ± 14	421 ± 54
	Area based equivalent diameter [µm] at 14 h	632 ± 26	847 ± 76	459 ± 36	527 ± 118
	Volume based equivalent diameter [µm] at 14 h	689	900	295	716
	Main pellet class at 20 h	class I	class II	class I	class I
Micromorphology	Number of spore cluster per pellet [-] at 14 h	1.64 ± 0.99	18.5 ± 17.55	1.13 ± 0.55	6.45 ± 4.81
	Total hyphal length per pellet [m] at 14 h	1.45 ± 0.86	3.57 ± 3.18	0.07 ± 0.08	1.37 ± 1.02
	Number of tips per pellet [-] at 14 h	8654 ± 4744	25,039 ± 23,222	599 ± 668	5871 ± 4711
	Number of branches per pellet [-] at 14 h	6258 ± 3465	18,414 ± 16,756	366 ± 445	6245 ± 4649
	Average branch length [µm] (0–20 h)	81 ± 23	75 ± 10	73 ± 11	94 ± 11
	Porosity [-] (0–20 h)	0.95 ± 0.02	0.95 ± 0.01	0.96 ± 0.01	0.96 ± 0.01
	Hyphal diameter [-] (0–20 h)	3.04 ± 0.06	3.07 ± 0.08	3.52 ± 0.38	3.24 ± 0.09

ΔCDW_max_*: Maximum increase in cell dry weight during cultivation (CDW_max_ - CDW_0h_) + talcum

In the case of larger pellets from the seed culture (1 g L^−1^), both STR1 and RMB1 were inoculated with approximately 200 pellets mL^−1^, with an initial average pellet diameter of 609 ± 31 µm (Fig. 3). Both reactors showed similar growth and glucose uptake patterns in the first 10 h (Fig. 2). However, glucose was almost consumed in STR1 after 12 h, whereas glucose ceased in RMB1 after 14 h, indicating faster substrate uptake in STR1 (Fig. 2). This is supported by the average volumetric glucose consumption rate, which was slightly higher in STR1 (0.60 g L^−1^ h^−^^1^) than in RMB1 (0.51 g L^−1^ h^−1^) (Table 1). During exponential growth, an average specific growth rate of 0.15 h^−1^ was achieved in the culture of the STR1 in comparison to the RMB1 (0.12 h^−1^, Table 1). Remarkably, the cultivation in the RMB1 had a higher biomass yield of 0.68 g g^−1^ compared to the STR1 (0.58 g g^−1^). These data suggest that in RMB1 a higher portion of the glucose was converted into biomass compared to the cultivation of STR1 (CDW_max_ of 6.73 ± 0.2 g L^−1^ vs. 5.81 ± 0.1 g L^−1^).

In terms of macromorphology, the average population developed differently under the two shear force regimes. In the STR1 cultivations, there was a three- to fivefold increase in pellet number concentration within the first 4 h (Fig. 3), despite no observable increase in the biomass concentration (Fig. 2). This, coupled with a decrease in the equivalent diameter by 100 µm (18%) (Fig. 3 and Supplementary Fig. S2a), suggests that the stirrer-induced shear forces led to pellet breakage at this early stage of cultivation. Initial pellet population in the STR1 differed significantly after just two hours (*p*-value < 0.001, Supplementary Fig. S3). While the pellet number concentration remained almost constant thereafter (Fig. 3), the proportion of dispersed mycelium increased towards the end of the cultivation (Supplementary Fig. S4), which indicates a loss of pellet integrity. In contrast in the RMB1 cultivations, pellet number concentration remained constant, while their diameter constantly increased by around 150 µm during the first 18 h of the batch cultivation (Fig. 3 and Supplementary Fig. S2a). In summary, the greatest difference in macromorphological development between shear force regimes occurred at the beginning of cultivation in the STR1, where the pellet population was shifted to smaller pellet diameters due to stirring. This resulted in a different initial pellet diameter composition between both cultivation systems (Fig. 3), which subsequently showed a different growth behaviour and macromorphological development.

For the smaller pellets from the seed cultures grown with 10 g L^−1^ of talcum, around 3000 pellets mL^−1^ were transferred to each bioreactor (STR10 and RMB10) featuring an initial pellet diameter of 234 ± 34 µm (Fig. 3 and Supplementary Fig. S2b). In terms of growth, STR10 and RMB10 cultivations resulted in highly comparable biomass yield (0.63 g g^−1^ in the STR10 and 0.62 g g^−1^ in the RMB10, Table 1) and CDW_max_ (Table 1 and Fig. 2). In RMB10 cultivations, glucose was consumed at a higher rate (0.64 g L^−1^ h^−1^, Table 1) compared to the RMB1 cultivations, while the highest volumetric glucose consumption rate among all cultivations was achieved in the STR10 (0.84 g L^−1^ h^−1^, Table 1).

With respect to macromorphology, both reactors showed similar trends in overall pellet diameter development and pellet number concentration when inoculated with the smaller pellet seed culture (Fig. 3 and Supplementary Fig. S2b). In STR10 cultivations, the pellet number concentration doubled after stirrer activation, and the pellet diameter declined by 24 µm (10%) within 2 h (Fig. 3 and Supplementary Fig. S2b). Nevertheless, compared to the larger STR1 pellets, the effect of the initial stirring to the pellet population was significant but marginal (*p*-value < 0.001, Supplementary Fig. S3). Likewise, in STR1, pellet diameter in STR10 cultivations increased mostly during exponential growth. The amount of dispersed mycelium was also highest in STR10 cultivations and increased compared to STR1 cultivations already during the growth phase (Supplementary Fig. S4). In RMB10, changes were evident within 2 h, with the population differing significantly from the seed culture (*p*-value < 0. 01, Supplementary Fig. S3) and, unlike STR10, shifting toward larger pellet diameters. Similar to the behaviour of larger pellets from RMB1 cultivations, the overall pellet diameter increased by an average of 240 µm in the RMB10 cultivations (Fig. 3 and Supplementary Fig. S2b).

In summary, the initial smaller pellets in the seed culture resulted in a more similar growth behaviour and macromorphological development between high and low shear regimes. This can be explained, as the effect of shear forces on the development of the pellet size distribution was more pronounced in populations with, in total, larger pellets (STR1 and RMB1) compared to populations with smaller ones (STR10 and RMB10). Comparing both systems, pellets in the RMB1 and RMB10 cultivations grew steadily into larger sizes than in all STR cultivations, in which the pellets reached rather similar final diameter ranges.

Next, the surface-to-volume ratio was calculated from the pellet diameters and pellet number concentration of the four cultivation conditions. When plotted against the average substrate uptake rates, a positive correlation was observed between a high surface-to-volume ratio and an increased uptake rate (Supplementary Fig. S5). Cultivations with a higher surface-to-volume ratio showed an earlier substrate depletion.

### Pellet diameter distributions and growth kinetics under high and low shear force conditions

It was further analysed whether there are obvious relations between the pellet size distribution in STR and RMB cultivations with seed cultures containing either 1 g L^−1^ or 10 g L^−1^ talcum and qualitative changes of specific rates (Supplementary Fig. S6).

With 1 g L^−1^ talcum, the initially larger pellet seed cultures demonstrated an almost constant specific growth rate (μ) throughout the core growth phase (6–12 h). The pellets in the STR1 cultivations had the most homogeneous diameter distribution, indicated by the smallest interquartile range (146 ± 10 µm, Supplementary Fig. S6). In RMB1, heterogeneity increased with pellet growth, characterised by an increase of the interquartile range from 267 ± 16 µm (0 h) to 346 ± 31 µm (10 h) (Supplementary Fig. S6). Higher peak substrate uptake rates were observable during the first four hours in STR1 and RMB1 cultivations, while the uptake rate was higher at STR1 cultivations, concomitantly to a reduced pellet diameter (Supplementary Fig. S6). A second, minor decrease in pellet diameter was observed in STR1 cultures after 12 h, which correlates with glucose limitation (Figs. 2, 3 and Supplementary Fig. S2a). In RMB1 cultivations, glucose limitation occurred later (Fig. 2), and the pellet diameter remained stable for a longer period (Fig. 3 and Supplementary Fig. S2a). A noticeable pellet diameter reduction was observed only when growth stopped, and glucose became completely depleted at the end of biomass growth (18 h). In contrast, cultures from 10 g L^−1^ talcum displayed a non-exponential growth pattern, as evidenced by an increasing specific growth rate over time (Supplementary Fig. S6). During the onset of the main growth phase (6–10 h), maximum diameter change in STR10 cultivations occurred (Fig. 3 and Supplementary Fig. S2b). As the pellets grew, the population became more heterogeneous, with the interquartile range expanding from 175 ± 24 µm at 0 h to 353 ± 20 µm at 20 h, effectively doubling the diameter range of the central 50% of the pellet population (Supplementary Fig. S6). While the pellet diameter was comparable between STR10 and RMB10 (Supplementary Fig. S2b), the size range of core 50% of the pellets was broader in RMB10 (402 ± 62 µm), making it the most heterogeneous population. Towards the end of the growth phase (10–12 h), distribution patterns stabilised in STR10 and RMB10 reactors for the remainder of the cultivation. Therefore, initially smaller pellet seed cultures led to faster initial growth, earlier growth decline and greater pellet heterogeneity, particularly in RMB10, before distributions stabilised towards the end of the growth phase.

### Product formation under high and low shear force conditions

**Fig. 4:**
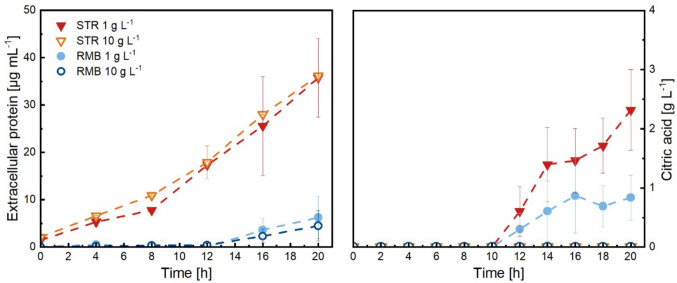
Extracellular protein and citric acid concentration were measured in the culture supernatant of duplicate STR and RMB bioreactor cultivations performed with an initial glucose (main carbon source) concentration of 8 g L^−1^. The values represent means calculated from biological replicates (**A** and **B**), and the error bars indicate the standard error

Extracellular protein titres were measured in all cultivations (Fig. 4). Highest protein concentrations were detected in both STR culture supernatant samples, while only a small amount was present in the RMB1 and RMB10 samples by the end of cultivation. Protein production in STR cultivations appeared to increase with glucose consumption. Secreted proteins are typically growth-related products (type I) according to Gaden’s classification (Gaden 1959). The highest protein levels were observed at the end of cultivations, with a total of 35 µg mL^−1^ of extracellular protein measured in STR1 and STR10 cultivations (5 µg mL^−1^ in RMB1 and RMB10). This was when the loss of pellet integrity marked by a decrease in pellet diameter (Fig. 3 and Supplementary Fig. S2a, b) and an increase in the proportion of dispersed mycelium (Supplementary Fig. S4) potentially contributed to the release of proteins into the medium. Thus, extracellular protein titres varied less between the different sizes of seed culture than between the two cultivation systems.

In contrast, seed culture history and pellet diameter significantly influenced citric acid production, a type II fermentation metabolite according to Gaden’s classification (Gaden 1959). These metabolites arise indirectly from reactions of the energy metabolism and typically follow an initial phase of microbial growth. No extracellular citric acid was detected in either the STR10 or RMB10 cultivations, where the pellets were small. However, in STR1 and RMB1 cultures, populations with larger pellet diameter, approximately 1 g L^−1^ of citric acid accumulated in the RMB1, and even twice as much in the STR1 cultures. Citric acid production began once the specific glucose consumption started to decline, though the specific growth rate had not yet reached zero (Fig. 4 and Supplementary Fig. S6). The strongest increase in production occurred within the following 4 h and 6 h for the STR1 and RMB1 cultivations, respectively. After glucose uptake ceased and growth stopped, the citric acid accumulation rate decreased in both reactor systems. The data showed that the shear force regime can have a considerable impact on both protein and citric acid production, whereas differences related to seed culture history were only observed for citric acid production. For the interpretation of the absolute values, it should be noted that all cultivations were performed with an initial glucose concentration of 8 g L^−1^, which is sufficient to support growth but not optimised for maximal production of secreted proteins or maximum accumulation of citric acid.

### Pellet class development and heterogeneity under high and low shear force conditions

**Fig. 5:**
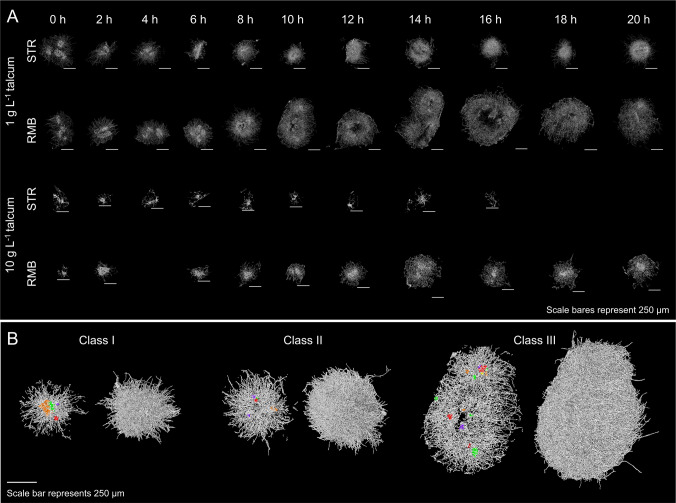
Synchrotron radiation-based microcomputed tomography was used to generate 3D data of *A.* *niger* pellets. **A** The development of the inner pellet architecture over the cultivation time is demonstrated by cross-sectional views for STR and RMB pellets. **B** Representative pellets were selected to visualise the classes of inner pellet architecture. Each pellet is shown from the outside and with a cross-sectional view of 25 µm through the centre of mass. Detected spores were clustered using the MATLAB function “dbscan” and shown 9 times enlarged. The images were created using VGSTUDIO MAX (version 3.2; Volume Graphics, Heidelberg, Germany)

As demonstrated previously in our work, the SR-µ-CT technique can be leveraged to determine the 3D structure of *A. niger* pellets down to the hyphal level (Müller et al. 2023). Accordingly, over 3500 pellets from STR and RMB runs (harvested from replicates A) were analysed. Representative pellet images of the different cultivation timepoints are given in Fig. 5A. First, the aim was to determine whether the volume-equivalent pellet diameters of the 3D data generally match with the area-equivalent pellet diameters of the 2D data (Supplementary Fig. S7a-d). For STR1 and RMB1 populations, the equivalent diameters measured with both methods were indeed very similar. In the STR10 and RMB10 populations, however, larger pellets (> 800 µm) from the STR10 population only matched to a limited extent with 3D data, while pellets from the RMB10 run were estimated to be overall larger compared to the 2D data. However, it was noted that the population dynamics in both cases were similar to those observed in the 2D data. Due to poor coverage, timepoint RMB10 4 h was excluded from further analysis. For STR10, data from 18 and 20 h are not available due to a file error.

**Fig. 6:**
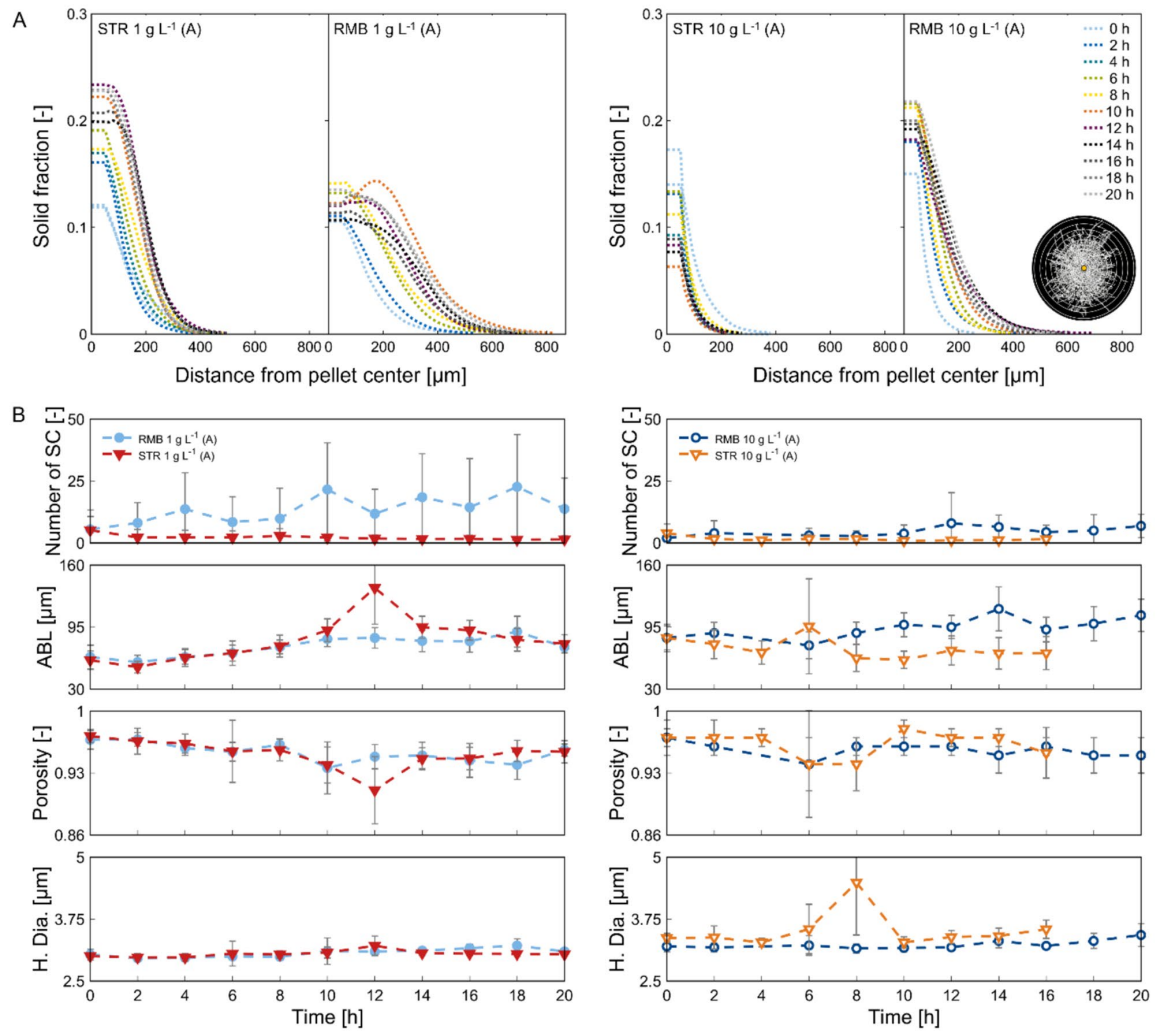
Population development from single pellet perspective calculated from synchrotron radiation-based microcomputed tomography data. **A** The solid fraction was plotted over the radial coordinate within the pellet. It was calculated for spherical shells with 15-µm width and an inner sphere of 50-µm radius and plotted as average solid fraction across the shells, as visually indicated with the example pellet at the right. Graphs with standard deviations are shown in Supplementary Fig. S8. **B** Mean number of spore clusters (SC), average branch length (ABL), porosity and hyphal diameter (H. Dia.) plotted over the cultivation time; the dashed lines serve to improve readability

In our recent study (Engelbert et al. 2025), a classification system of coagulative pellets based on their inner architecture was introduced and is summarised in the introduction. Figure 5B represents 3D images of classes I to III that were found in populations in this study. The solid fraction (= the amount of hyphae and embedded talcum) at the pellets mass centre, along with the calculated number of spore clusters within the pellets (Fig. 6), was shown to be reasonable indicators for estimating pellet classes within a population. Note that the number of visible spore agglomerates may differ from the number of spore clusters determined in three dimensions.

For the pellet population obtained from seed cultures with 1 g L^−1^ talcum, pellets of class I to III were observed and used for inoculating the bioreactors, with the main share consisting of pellets of class II, counting 5.33 ± 0.23 spore clusters per pellet. The solid fraction at the pellets’ mass centre was 0.11 ± 0.01. In the STR1, pellet breakage assumed to result from stirring shifted the population from predominantly multi-spore agglomerate pellets (class II) to single-spore agglomerate pellets (class I) within the first two hours of cultivation. This is indicated by a reduced number of spore clusters per pellet (2.25 ± 3.19) and change in the overall trend of the solid fraction (Fig. 6A). This is also reflected in the pellet images (Fig. 5A). The solid fraction increases evenly afterwards, indicating that the pellets grow very uniformly radially with mostly one centred spore cluster (Fig. 6). A reduced solid fraction in the outer part of the pellet (~ 180–400 µm) (Fig. 6A) was observed after growth ceased (µ = 0) at 16 h (Supplementary Fig. S6). Together with the loss of pellet integrity observed by an increase in dispersed mycelia (Supplementary Fig. S4), this could indicate abrasion of the outer hyphae.

In contrast to the STR1, the RMB1 pellets are characterised by less dense and overall fluffier pellet architecture, featuring longer outward hyphae (Fig. 5A). The visually looser structure is supported by a lower solid fraction at the mass centre, with a maximum of approximately 0.15 at 8 h (Fig. 6A). The number of spore clusters per pellet increases slightly during the cultivation, being a strong indication that some pellets fuse, especially at the beginning of the growth phase (4 h), thereby increasing the share of class III pellets. This trend is supported by an increase in pellet diameter and a slight decrease in pellet number concentration from 4 to 6 h (Fig. 3 and Supplementary Fig. S2a). The high standard deviation of the spore clusters (Fig. 6B) reflects the high heterogeneity in pellet diameter of this population (Supplementary Fig. 2a and S6). Within the first 8 h, pellets from the RMB1 cultivations show an evenly distributed increase of hyphal mass in all parts of the pellets (Fig. 6A). In contrast to the STR1, in RMB1, the hyphal fraction distributions were less defined after glucose limitation (14 h). This can be attributed to the high variety of different pellet architectures in the RMB1 population, represented by only a low number of analysed pellets (*n* < 40 per timepoint).

However, pellets from seed cultures with 10 g L^−1^ talcum developed differently under the two shear stress regimes. The seed culture consists mainly of pellets with very few spore clusters 2.96 ± 1.39 and a solid fraction of 0.15 ± 0.02 at the mass centre (Fig. 6) and can therefore be considered mainly as class I pellets. In the STR10 culture, more loosely structured macromorphologies were formed, consisting of a mixture of small pellets and clumps with low overall density (Fig. 5A). The solid fraction at the mass centre has the highest value at 0 h and decreases continuously to the lowest values < 0.1 of all cultivations at 14 h. Similar to the STR1 pellets, the activation of the stirrer had a large impact on the single pellet morphology of STR10 indicated by a change of the solid fraction trend (Fig. 6A). Additionally, the number of spore clusters was reduced by more than half after stirrer activation (Fig. 6B) while the pellet diameter decreased, too (Fig. 3 and Supplementary Fig. S2b). Interestingly, the increase in pellet diameter during growth (6–12 h), visible in Fig. 3, is not reflected by the solid fraction of the 3D data. A reason could be a reduced capturing of pellets with larger diameters or a potential higher share of dispersed mycelium (Supplementary Fig. S4) that in general cannot be captured by SR-µ-CT. Pellets from the RMB10 culture were morphologically similar to those of the STR1 culture but appeared less dense and fluffier (Fig. 5A). Likewise, they show the same uniform outward growth, starting mostly from a central spore agglomerate with an even increase in solid fraction. The number of spore clusters per pellets increased slightly during growth to 7.97 ± 12.29 (12 h) in RMB10, but stayed on a lower level compared to the RMB1 seed culture.

Therefore, it could be concluded that the two distinct seed culture populations have developed differently on a micromorphological level under the high and low shear force regimes. While in the STR cultivations, pellet populations with mainly multiple spore agglomerates (class II) were transformed to class I pellets, class I start populations were hindered from exhibiting a dense hyphal structure. In the RMB cultivations, however, the initial inner architecture was maintained. Also, the low shear forces in the RMB seemed to promote the fusion of pellets through hyphal entanglement and therefore increased the share of class III pellets in the overall pellet population.

In addition to analysing the populations on a micromorphological level, various other key parameters were computed by the SR-µ-CT as shown in Fig. 6B, Supplementary Fig. S9 and Table 1. Further data are provided in Supplementary Material 1. The pellet total hyphal length, number of tips and number of branches per pellet increased over cultivation time in a similar range as the pellet diameter (Supplementary Fig. S9). At the end of the growth phase (14 h), a pellet in the STR1 with a diameter of 689 µm had in total 1.45 ± 0.86 m of hyphal length counted 8654 ± 4744 tips and 6258 ± 3465 branches, while at the same time for RMB1 a diameter of 900 µm, 3.57 ± 3.18 m hyphae, 25,039 ± 23,222 tips and 18,414 ± 16,756 branches per pellet were measured. For the smaller pellets of the 10 g L^−1^ seed cultures, numbers at 14 h were as follows: 0.07 ± 0.08 m hyphae, 599 ± 668 tips and 366 ± 445 branches for pellets with a median diameter of 295 µm in STR10 and 1.37 ± 1.02 m hyphae, 5871 ± 4711 tips and 6245 ± 4649 branches for pellets with a diameter of 716 µm in RMB10. The average branch length (ABL), describing the length between two branching points or a branching point and a tip (Müller et al. 2023), is given in Fig. 6B and shows no substantial changes over time. The averaged ABL value over 20 h is in a similar range for all the conditions: 81 ± 23 µm, 75 ± 10 µm, 73 ± 11 µm and 94 ± 11 µm for STR1, RMB1, STR10 and RMB10 populations, respectively (Fig. 6B, Table 1). This is an indication of a constant apical growth behaviour and branching pattern. Interestingly, while the local distribution of hyphal mass differed between pellets formed under the two seed culture conditions and shear stress regimes (Fig. 6A), the average pellet porosity (0.95 ± 0.02, 0.95 ± 0.01, 0.96 ± 0.01 and 0.96 ± 0.01 for STR1, RMB1, STR10 and RMB10, respectively) remained largely constant throughout the cultivation period (Fig. 6B, Table 1). Also, hyphal diameter (3.04 ± 0.06, 3.07 ± 0.08, 3.52 ± 0.38 and 3.24 ± 0.09 for STR1, RMB1, STR10 and RMB10 populations, respectively) stayed mostly unchanged (Fig. 6B, Table 1). It has to be mentioned that in direct comparison, the average hyphal diameter was overall slightly greater in cultivations with 10 g L^−1^ of talcum at the seed compared to those with 1 g L^−1^. However, the hyphal diameters are in the same range as reported in previous studies for *A. niger* (Schmideder et al. 2019a; Müller et al. 2023). Taken together, general micromorphological mycelial characteristics of *A.* *niger* such as branching frequency, overall pellet porosity and hyphal cell wall thickness were rather constant in both shear force regimes and thus likely under genetic self-control.

## Discussion

Since morphology and productivity are closely linked in filamentous microorganisms, understanding how the cultivation system impacts the morphology of a specific population holds significant importance for strain and process optimisation. In the present study, the influence of high or low shear forces, using two different reactor systems (RMB vs STR), on a defined seed population was investigated. While the importance of stirrer-induced shear effect on the overall macromorphology of filamentous fungi has been explored by many (Grimm et al. 2005; Lin et al. 2010), the morphological development under low shear force regimes in shaken, but nevertheless controlled cultures has been rarely investigated, and mainly by us (Kurt et al. 2018; Kheirkhah et al. 2023). It has been reported further that lower agitation speeds in an STR can inhibit pellet growth due to oxygen limitation in the bulk liquid phase due to insufficient gas–liquid mass transfer. This makes it impractical to operate these reactors at low shear forces without adversely affecting pellet morphology while still ensuring sufficient oxygen supply (Li et al. 2002).

To control the initial diameter range of the population, the seed culture was either supplemented with 1 g L^−1^ or 10 g L^−1^ talcum microparticles. This approach ensured the integration of talcum within the pellets, preventing the presence of free talcum while simultaneously allowing control over pellet diameter. Additionally, this method enabled monitoring of the seed train. This study primarily focused on the effect of shear forces on the populations in the two bioreactor systems, and dissolved oxygen (DO) levels were consistently maintained above 40% in all cultivations (Supplementary Material 1), ensuring that oxygen limitation caused by insufficient gas–liquid mass transfer into the culture medium can be excluded. However, despite maintaining the same aeration rate in both reactors, differences in fluid dynamics lead to variations in the k_L_a value. While the impact of oxygen transfer rate is generally less pronounced than that of shear forces (both by stirrer and aeration), it should not be overlooked, as it can still influence the pellet morphology of *A. niger* spp., especially on the microlevel (Casas López et al. 2005; Lin et al. 2010).

**Fig. 7:**
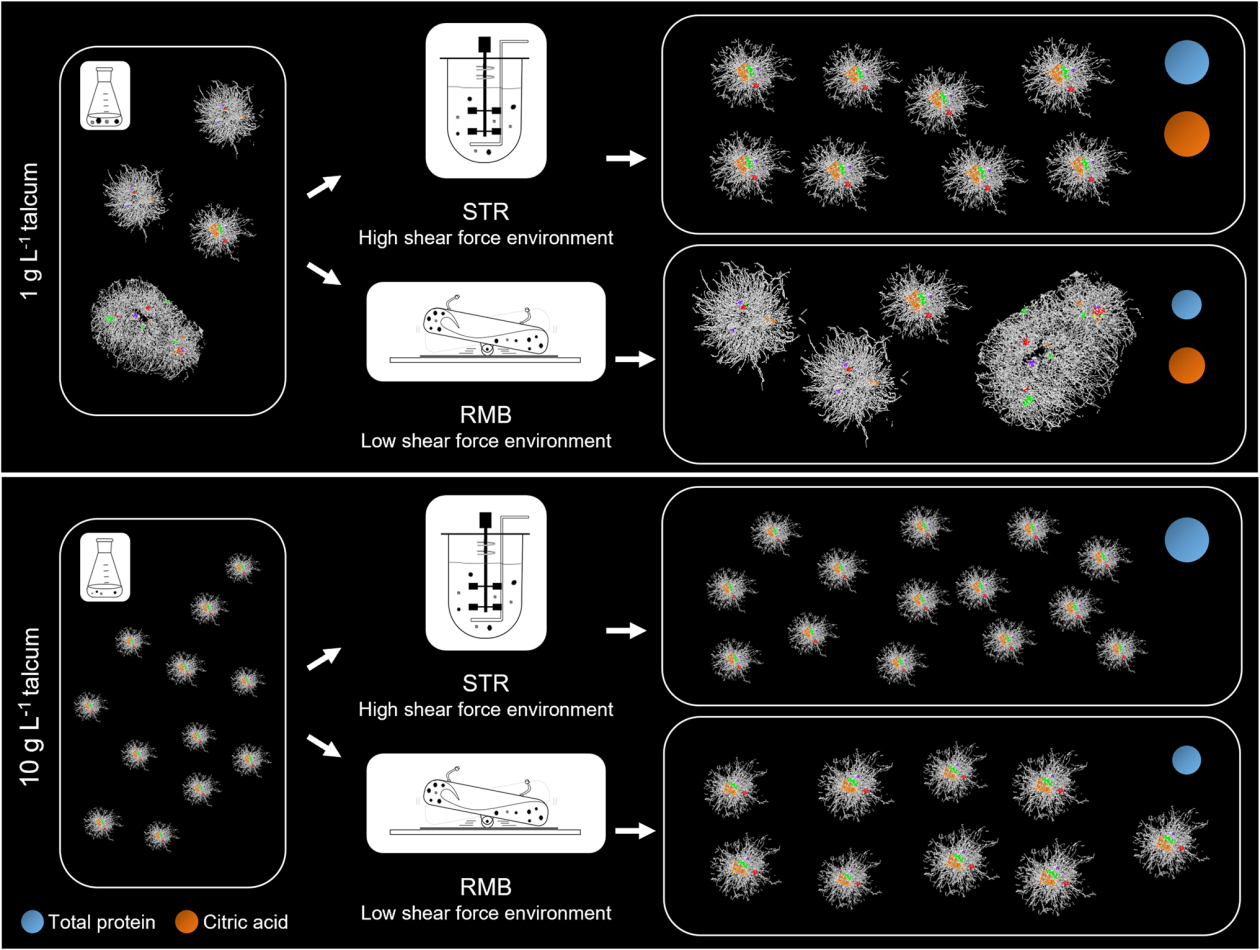
Graphical summary of the influence of high and low shear force conditions on two different pellet seed culture populations. Cultures in which protein or citric acid were measured in the supernatant are marked by blue and orange circles, respectively

The morphological development of the culture was then compared in the two bioreactor systems on both macro- and micromorphological levels, alongside the growth parameters and product formation. The results of this study are summarised in Fig. 7. In general, a lower talcum concentration resulted in the formation of fewer but larger pellets, most of which encompass multiple spore clusters in the seed culture (Fig. 6B). This population (1 g L^−1^ talcum) showed a more distinct growth behaviour across the different reactor systems. This can be explained primarily by the different number and diameter of the particles in the two systems, which resulted in a varied surface-to-volume ratio. For the pellet population with initially smaller inoculated pellets (seed culture with 10 g L^−1^ talcum), a more similar growth behaviour and morphological development were observed under high and low stress regimes. Apart from that, RMB cultivation pellets were always fluffier with longer outwards hyphae compared to the STR cultivation pellets. It has been previously reported that lower power input and thus a regime of lower shear forces lead to the formation of fluffier pellets (Böl et al. 2021).

For larger pellets, two distinct shear-related effects on the overall morphology were identified. Firstly, the pellet diameter was made more uniform in the high shear stress system, leading to pellets with a centralised spore agglomerate, which only increased in diameter during fermentation. The effect of shear force on the breakage of larger pellets from STR cultures was evident right after stirrer activation (data not shown), leading to an increase in the pellet number concentration and a decrease in their median equivalent diameter. Secondly, it was observed that after glucose limitation under higher shear forces, hyphal network integrity could not be maintained, as evidenced by a higher proportion of dispersed mycelium in the medium compared to a low shear force regime. In terms of heterogeneity, pellets in the RMB had a higher variation in the number of spore clusters per pellet, leading to an overall more heterogeneous pellet population. It is important to note that because the seed culture population is inherently heterogeneous, the rocking-motion does not significantly alter the morphology; rather, it maintains this heterogeneity, allowing the pellets to grow without substantial morphological changes provided that sufficient substrate is available. This assumption is supported by an increase in the median diameter while maintaining a constant pellet number concentration. Larger pellets in the STR1 cultures, on the other hand, formed the most uniform culture among all. Due to shear forces, pellets could not surpass a certain diameter, therefore ending up more homogenous. This size-limiting effect has been described previously for pelleted inoculum under high shear stress STR cultivation (500–800 rpm) (Paul et al. 1999). Additionally, glucose availability per pellet presumably contributes to pellet diameter restriction, given a higher pellet number concentration in STR1 compared to RMB1 cultivations.

Pellets originating from the seed culture containing 10 g L^−^^1^ talcum were initially smaller and underwent less drastic morphological changes in the reactor systems compared to the larger ones. Predominantly composed of a single spore agglomerate, these pellets mainly maintained their structural integrity even under the higher shear conditions of the STR, although a slight increase in pellet number concentration was still observed. In contrast, these smaller pellets occasionally fused in RMB, indicated by a decrease of pellet number concentration and increase of pellet size by 60 µm within the first 4 h, resulting in the most heterogeneous macromorphology (diameter) among all conditions. This increased heterogeneity likely stems from their small initial size and high pellet number concentration, which allowed for a broader diameter range during cultivation. This distinction should be considered when selecting a cultivation system, since heterogeneity can be beneficial for an improved enzyme portfolio and overall production robustness (Lyu et al. 2023).

As macromorphological development was followed, micromorphological development of pellet architecture was also assessed using solid fraction and the number of spore clusters. All growth-related classes I, II and III were identified in this study, which develop from one or more spore agglomerates or mature pellets. Results from 2D image analysis of this study as well as other studies (Müller et al. 2022) show the breakage of pellets, resulting in temporary structures that can neither be assigned to any of these three classes nor to dispersed mycelia. This collection of pellet fragments is herein referred to as the process-oriented, provisional ‘class IV (broken pellets)’ to facilitate discussion while acknowledging that a systematic definition and quantitative characterisation would require further dedicated study. These fragments are likely associated with high shear forces and may transition to class I or II through subsequent growth, as well as potentially serve as precursors for class III. Such fragment-derived pellet formation has been reported in our previous work (Engelbert et al. 2025).

In terms of physiology, the growth and substrate uptake rates were slightly higher in STR compared to the corresponding RMB, regardless of the concentration of talcum in the seed culture. The highest specific substrate uptake rate was observed in STR10 pellets, which also had the smallest median pellet diameter and, consequently, the highest surface-to-volume ratio. Similar diameter-dependent effects on glucose consumption were also observed in our previous shake flask study, where cultures with higher pellet number concentrations and populations with smaller diameters consumed glucose more rapidly (Engelbert et al. 2025).

Interestingly, the highest maximum biomass was reached in RMB1 cultures, which were also characterised by the largest median pellet diameter (Table 1). This implies that these larger pellets could grow more in size under low shear stress regime. One might argue that, despite the absence of oxygen limitation in the culture medium, growth hindrance due to mass transfer limitations within large pellets raises questions about the applicability of the RMB system. Recent studies, however, showed that oxygen and substrate diffusion rate in the pellet is mainly affected by hyphal fraction (Hille et al. 2009; Schmideder et al. 2021). Despite the significantly larger average pellet size in RMB, the pellets’ average porosity is similar to the ones from STR. What differs in RMB, though, is the distribution of hyphal mass over the pellets. By maintaining initial (multi-spore agglomerate) pellet architectures, the less dense pellet centre structure in the RMB may facilitate better transfer of nutrients and oxygen. These features could contribute to a slightly higher metabolic efficiency, as indicated by the biomass yield.

So far, it was outlined that in STR presumably less metabolic resources were converted to biomass in contrast to the RMB. These resources might have been used to secrete proteins as significantly higher protein levels were measured in STR compared to RMB during the growth phase. The inverse relationship between biomass formation and protein secretion, which rely on the same metabolic resources, has been previously demonstrated for *A. niger* (Melzer et al. 2007). Consistent with this, it was recently shown that under oxygen-limited conditions in shake flasks, populations with the lowest biomass had the highest protein secretion (Engelbert et al. 2025). Those pellets, however, were characterised by the largest diameter. Apart from the metabolic resources, shear stress may also enhance protein secretion as both STR cultivations showed significantly higher extracellular protein accumulation. This observation is in line with the hypothesis that secretory vesicles, which are required for maintaining cell wall integrity, are also involved in protein secretion and may therefore represent a shared and potentially limiting resource (Kunz and King 2022). In this context, previous studies have shown that cell wall stress, which activates the cell wall integrity (CWI) pathway, leads to transcriptional upregulation of genes involved in vesicle trafficking and the secretory machinery (Fiedler et al. 2014). However, experimental data by Kunz and King (2022) suggest a more complex relationship. They quantified fluorescently tagged vesicles and glucoamylase distribution in *A. niger* under different hydrodynamic shear conditions in a microfluidic chamber. Their results indicate that neither vesicle abundance nor protein secretion scale linearly with increasing shear stress but rather exhibit an optimum behaviour depending on the applied hydrodynamic conditions. Therefore, while shear stress may influence protein secretion, the observed differences in this study are more consistently explained by changes in biomass formation and resource allocation.

The influence of pellet macromorphology on citric acid accumulation in *A. niger* cultivations has been recognised for many years (Gómez et al. 1988; Papagianni et al. 1994; Paul et al. 1999). Consequently, the use of seed cultures to ensure a controlled morphology in the main fermentation is considered a common practice (Papagianni and Mattey 2006). In this study, citric acid production was observed only in cultures supplemented with 1 g L^−1^ talcum in the seed culture. This may be linked to differences in specific growth and substrate uptake rates, as both bioreactor systems showed a comparable growth pattern when derived from the same seed culture. This could suggest that seed culture history, including initial pellet diameter, morphology and physiological state might have a stronger influence on growth than the actual pellet diameter at any given time point during cultivation. Measurable amounts of citric acid were detected as the phase of intense growth ended. Notably, citric acid production is known to occur under substrate-limiting conditions but requires active growth. Following the growth patterns of all cultivations, the window of time with the required metabolic state might have been too short for citric acid production in 10 g L^−1^ cultures. The higher production in the STR1 compared to the RMB1 could accordingly be attributed to increased substrate limitation within the more densely compacted core of pellets in STR1. For higher citric acid accumulation, stable pellets/clumps cultures with small sizes (< 0.5 mm diameter) have been previously described as beneficial (Gómez et al. 1988; Papagianni 2007; Max et al. 2010).

In a population, it is assumed that pellets of different sizes take on different tasks, e.g., as the main enzyme producer or for the formation of secondary metabolites such as anti-stress factors (Lyu et al. 2023). The ability to control the population size via the choice of seed culture and shear force regime towards a more homogeneous or heterogeneous direction could thus be advantageous for optimising product yield or population stability. However, most of the previous studies have focused on the effect of process conditions on cultivations starting from spores (Kelly et al. 2004; Lin et al. 2010; Wucherpfennig et al. 2012; Kurt et al. 2018). Alternatively, some studies opted for the conventional seed culture preparation method without actively controlling the morphology prior to the main cultivation (Fazenda et al. 2010; Maumela et al. 2021). A potential issue that may arise there is the transferability of these findings across scales. Only a limited number of studies have systematically tracked the development of a specific seed culture within the bioreactor system (Lu et al. 2015; Waldherr et al. 2023). Waldherr et al. (2023) conducted comprehensive research on the impact of hydromechanical stress using various stirrer types on harvested pellets of *A. niger*. Their study was conducted in a diluted media where fungal growth was not a contributing factor, and the sole focus was rather on the shear-induced pellet breakage. That being said, as far as our knowledge extends, no study has yet combined the impact of shear forces in controlled bioreactor cultivations and the cultivation history (pellet with defined characteristics at inoculation) on the process response in rocking vs. stirred cultures.

## Conclusion

This study provides the first scalable framework for optimising filamentous bioprocesses through population heterogeneity engineering. By inoculating defined seed populations into a STR and a RMB, we investigated the combined influence of mechanical stress and initial pellet architecture on the micro- and macromorphology, growth behaviour and metabolite production of *A. niger*. This demonstrates, for the first time, the following of seed culture development at both two- and three-dimensional levels in various shear-induced environments. Our results show that classical STR environments restrict pellet sizes and enforce a homogenising effect, both at the population level and within the internal architecture of individual pellets. This is primarily attributed to shear-induced pellet disintegration leading to compact, centralised growth units. In contrast, low-shear force regime allows heterogeneous seed cultures to retain their diversity and support the development of larger pellets with a fluffier structure. Higher levels of extracellular protein, however, were observed in STR with smaller and more compact pellets. Citric acid production, on the other hand, seemed to be related to seed culture history.

## Supplementary Information

Below is the link to the electronic supplementary material.

ESM 1 (PDF 2.84 MB)

ESM 2 (XLSX 233 KB)

## Data Availability

The data sets used and/or analysed during the current study are available from the corresponding author on reasonable request.
