# Supplementary material for: Understanding pellet population heterogeneity of Aspergillus niger in stirred tank and rocking motion bioreactors
Source: Appl Microbiol Biotechnol. 2026 May 8;110(1):140. doi: 10.1007/s00253-026-13822-0 (PMC13156162; doi:10.1007/s00253-026-13822-0)
Supplement: Supplementary file 1 — (PDF 2.84 MB) [file 253_2026_13822_MOESM1_ESM.pdf]

Applied Microbiology and Biotechnology

Supplementary Information to

Understanding pellet population heterogeneity of *Aspergillus niger* in stirred tank and rocking motion bioreactors

Karin Engelbert*[1], Tolue Kheirkhah*[2], Charlotte Deffur[3]; Fangxing Zhang[3], Henri Winter[3], Timothy Cairns[1], Sascha Jung[1], Heiko Briesen[3], Peter Neubauer[2], Stefan Junne[2,4], Vera Meyer[1]§

*Authors contributed equally

§ Corresponding author

[1]Technische Universität Berlin, Institute of Biotechnology, Chair of Applied and Molecular Microbiology, Gustav-Meyer Allee 25, D-13355 Berlin, Germany

[2] Technische Universität Berlin, Institute of Biotechnology, Chair of Bioprocess Engineering, Ackerstraße 76 ACK24, D-13355 Berlin, Germany

[3] Technical University of Munich, School of Life Sciences Weihenstephan, Chair of Process Systems Engineering, Gregor-Mendel-Straße 4, 85354 Freising, Germany

[4] Aalborg University, Department of Chemistry and Bioscience, Niels Bohrs Vej 8, DK-6700 Esbjerg, Denmark

Correspondence:

Vera Meyer, E-mail: vera.meyer@tu-berlin.de

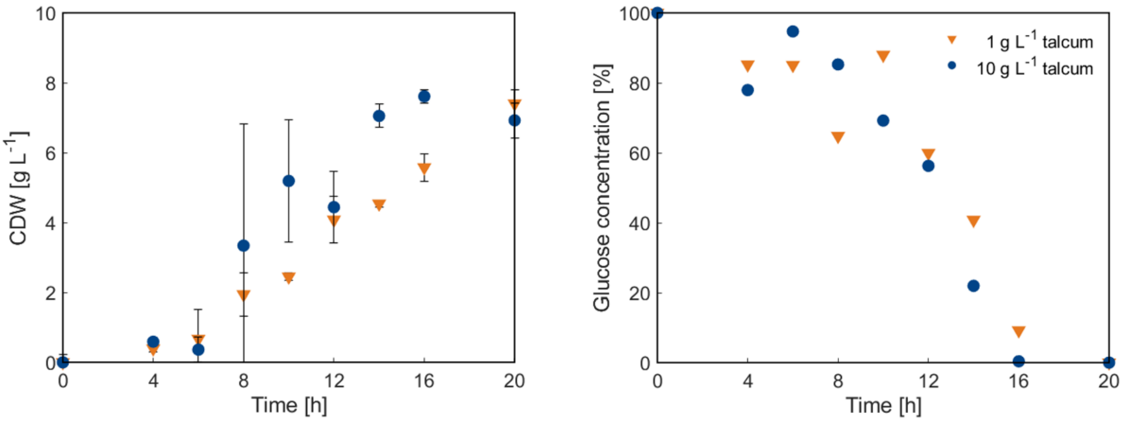

Fig. S1 Growth behaviour of seed cultures grown with 1 g L^{-1} or 10 g L^{-1} talcum in CM media. One shake flask was harvested for each time point inoculated with the same spore solution of 5 $\times 10^6$ spores mL^{-1}. To determine the cell dry weight (CDW), the flasks were sampled three times and the percentage by weight of the talcum was subtracted from the biomass (left). Normalised glucose concentration (right). Data originally published in Engelbert et al., 2025; adapted here.

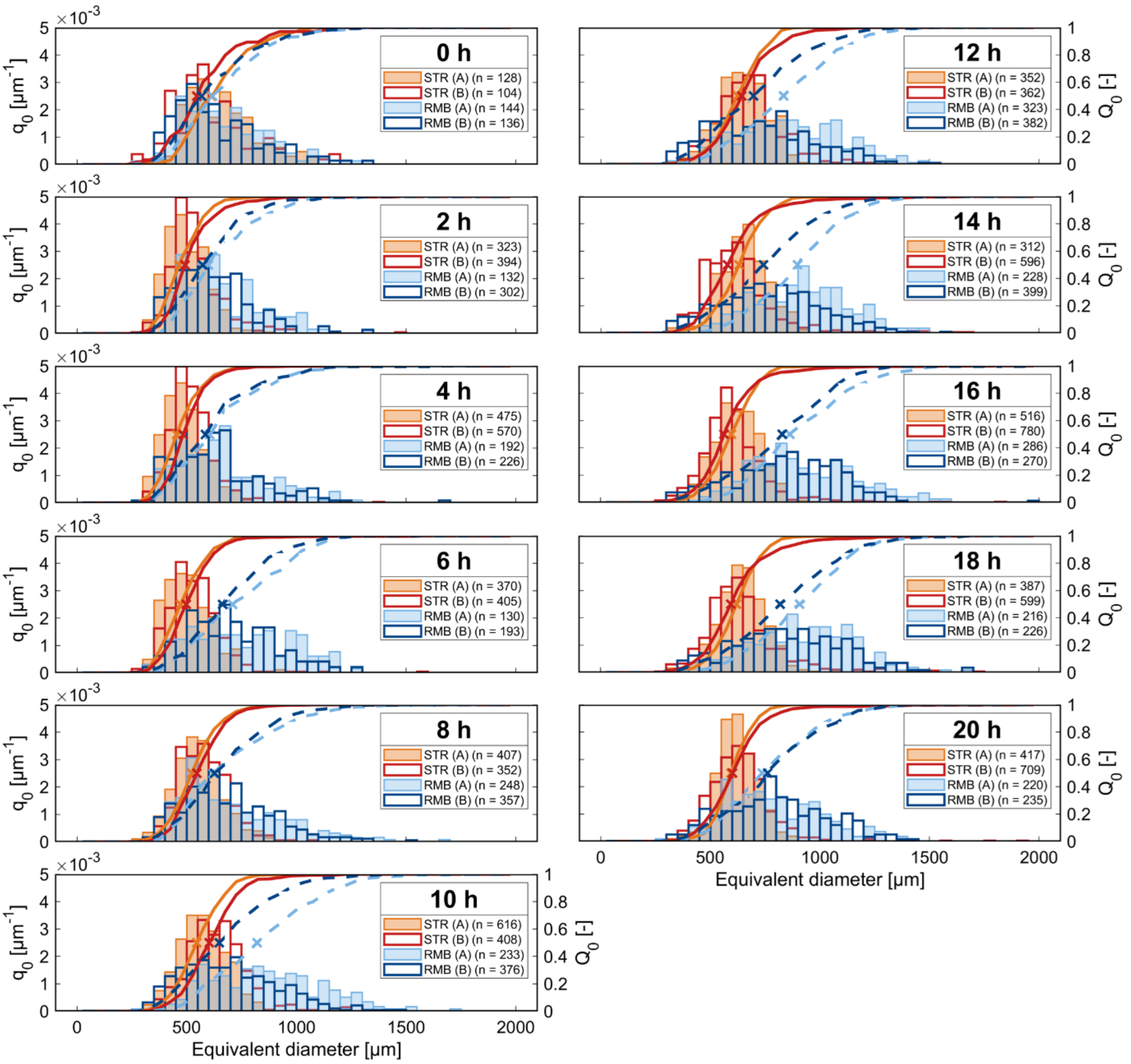

Fig. S2a Comparison of equivalent pellet diameter distributions obtained from 2D (area based) image analysis for the 20 h batch cultivation of STR and RMB 1 g L^{-1} talcum seed culture. Histograms represent normalized frequency distributions (q_0) with a bin size of 50 µm, while the solid (STR) and dotted (RMB) lines show the corresponding cumulative distributions (Q_0). The "X" indicates the median diameter and (*n*) indicates the number of analysed pellets.

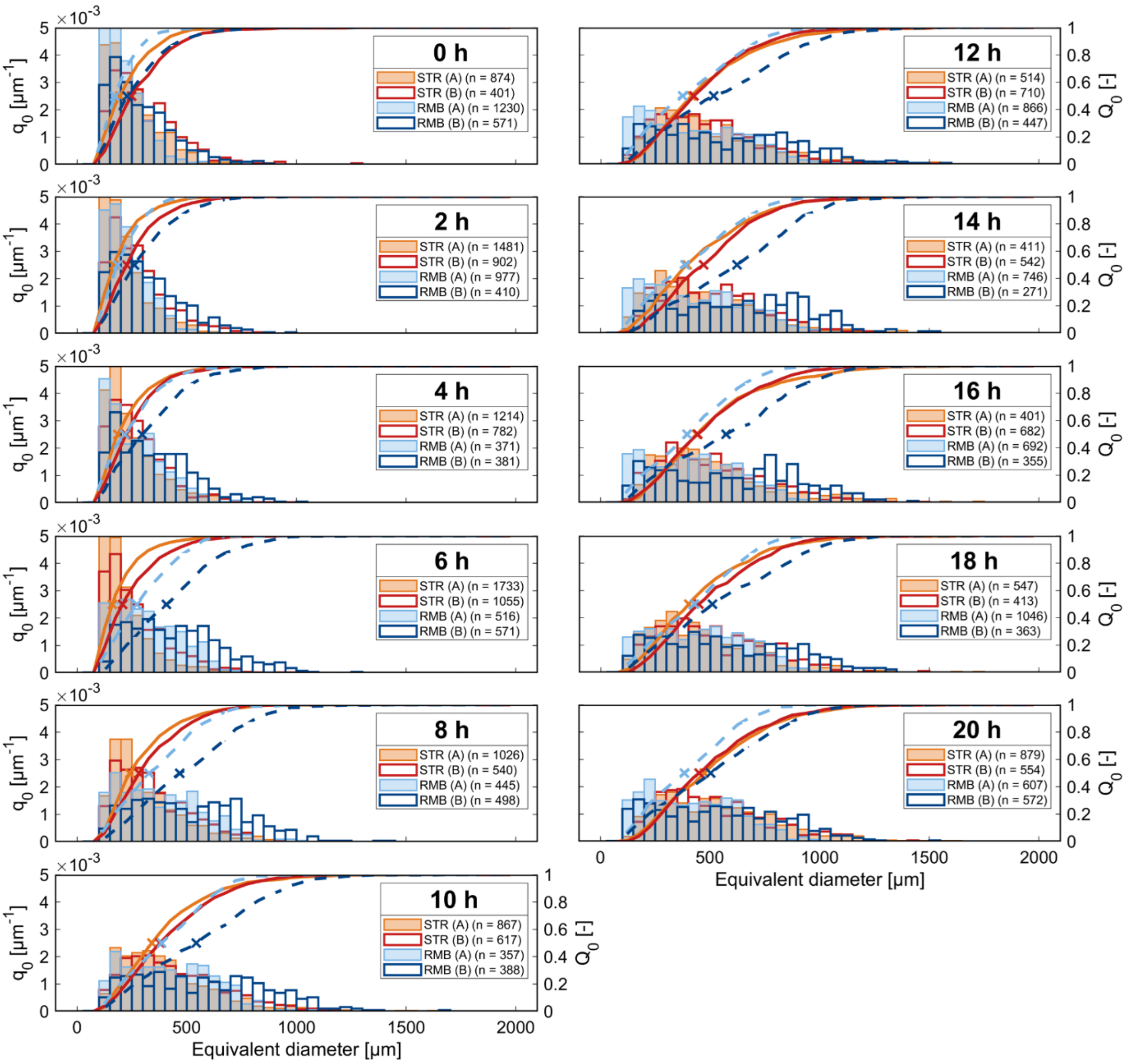

Fig. S2b Comparison of equivalent pellet diameter distributions obtained from 2D (area based) image analysis for the 20 h batch cultivation of STR and RMB 10 g L^{-1} talcum seed culture. Histograms represent normalized frequency distributions (q_0) with a bin size of 50 µm, while the solid (STR) and dotted (RMB) lines show the corresponding cumulative distributions (Q_0). The "X" indicates the median diameter and (*n*) indicates the number of analysed pellets.

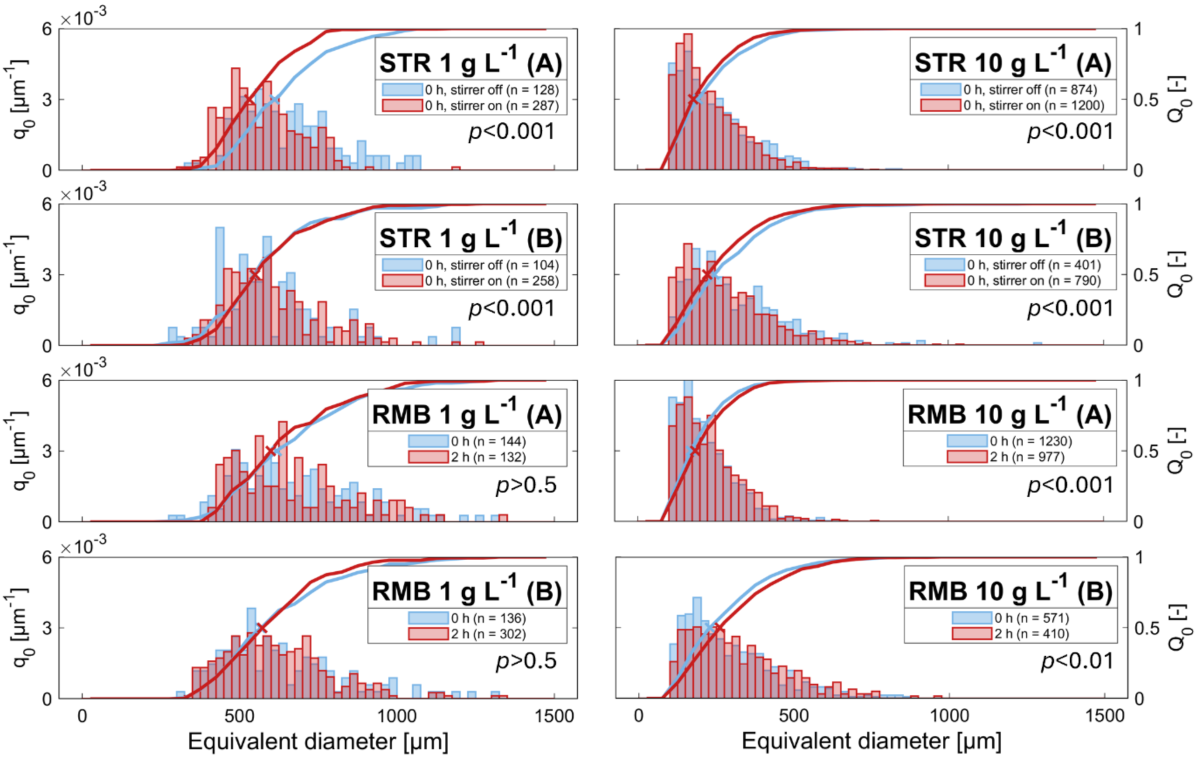

Fig. S3 Comparison of pellet diameter distributions derived from 2D image analysis at 0 h and 2 h of cultivation time, to assess the impact of stirrer activation on STR and RMB systems for each condition. Histograms represent normalized frequency distributions (q_0) with a bin size of 25 µm, while the lines show the corresponding cumulative distributions (Q_0). The "X" indicates the median diameter and (*n*) indicates the number of analysed pellets. The difference of the respective populations were analysed using the Mann–Whitney *U* test. The null hypothesis "The two populations have the same distribution" was rejected if the *p*-value was <0.05. *p*-values of the compared populations are indicated in the graphs.

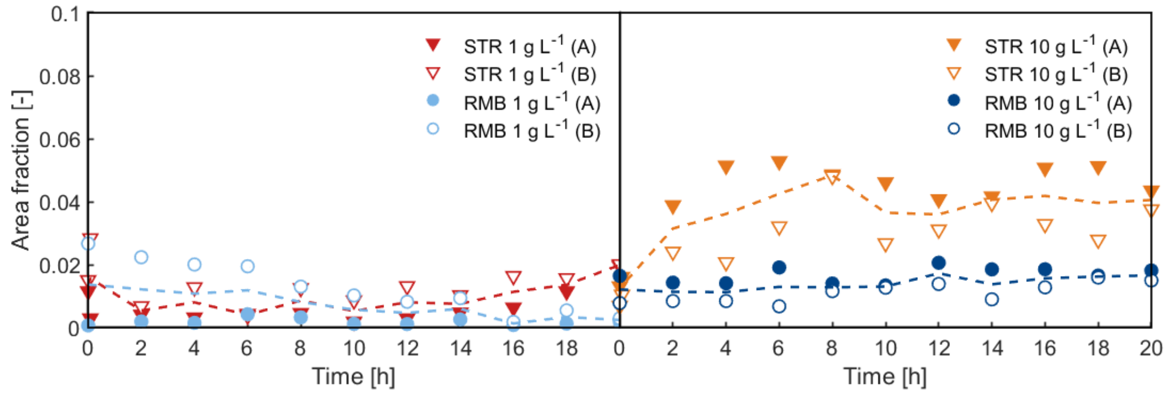

Fig. S4 Dispersed mycelium measured during 20 h batch cultivation by 2D image analysis. The dashed lines show the mean value of each replicates A and B. Measurement uncertainty may arise from surface artefacts such as scratches on the glass plate, which become more prominent when biomass coverage is low.

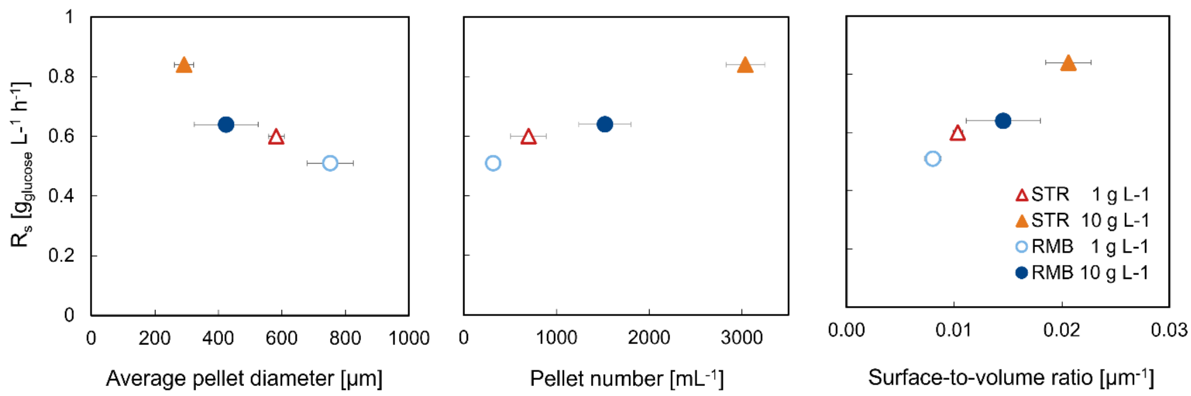

Fig. S5 Plotting the substrate uptake rate (R_S) (see Table 1) against the average pellet diameter, pellet number concentration and surface-to-volume ratio during exponential growth phase. The average pellet diameter was conducted by calculating the median of replicates A and B over the growth phase each and calculating the mean of these values. Values for the pellet concentration were conducted similar. Error bars represent the standard deviation of replicates A and B. The total surface area was conducted by calculating the spherical surface volume for the respective average pellet diameter and scaled with the measured pellet number concentration.

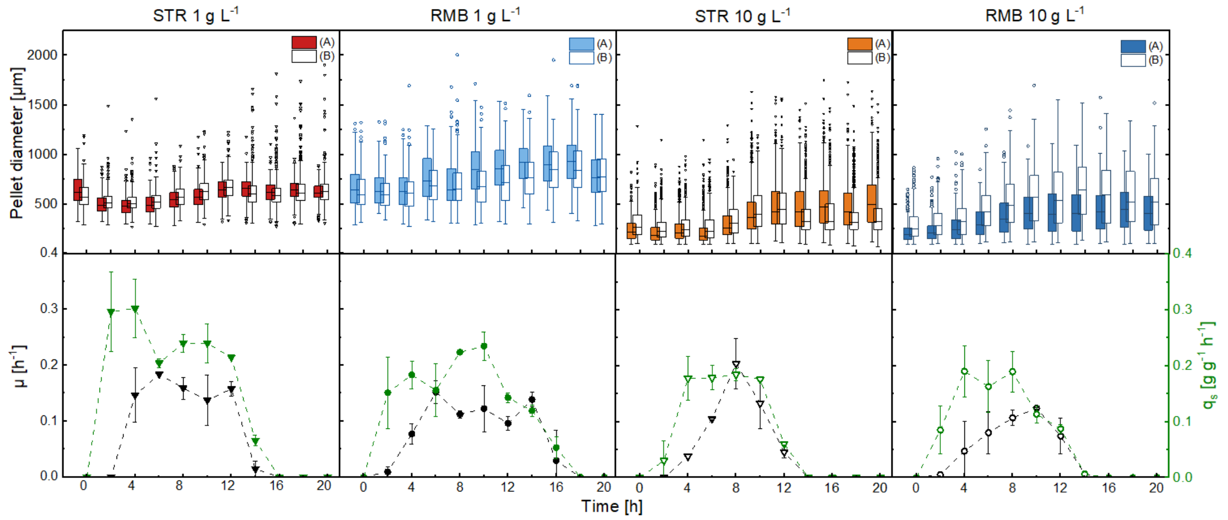

Fig. S6 Pellet diameter distribution and growth characteristics of two pellet seed cultures in a stirred tank and rocking-motion bioreactor system. Runs were performed in duplicates in a 20 h batch cultivation. Box plots (top) showing population heterogeneity based on the area equivalent pellet diameter. Apparent local values of the specific growth rate (μ) and specific substrate uptake rate (q_s) were estimated using equations S1–S3 between two following timepoints to illustrate qualitative trends during cultivation. For μ and q_s the mean values of duplicate runs (A, B) are given, error bars represent the standard error.

Calculation of specific rates

Time-dependent specific growth rates (μ, h^{-1}) and specific substrate uptake rates (q_s, g g^{-1} h^{-1}) were determined for discrete time intervals between consecutive sampling points (t_i and t_{i+1}) with logarithmic linearization:

$$\mu_i = \frac{\ln(X_{i+1}) - \ln(X_i)}{t_{i+1} - t_i} \qquad \text{(S1)}$$

where X_i and X_{i+1} represent the biomass concentrations (g L^{-1}). The specific substrate uptake rate q_s was calculated assuming a linear or almost linear growth behavior in the respective time interval, utilizing the mean biomass ($\bar{X}_{interval}$, gL^{-1}) to account for biomass accumulation during the interval:

$$q_{s,i} = \frac{S_i - S_{i+1}}{\bar{X}_{interval}(t_{i+1} - t_i)} \qquad \text{(S2)}$$

with $\bar{X}_{interval}$ defined as:

$$\bar{X}_{interval} = \frac{X_{i+1} + X_i}{2} \qquad \text{(S3)}$$

and S_i, S_{i+1} denoting the respective substrate concentrations (g L^{-1}).

Maximum specific rate in the manuscript (μ_{max}) was determined to compare the culture performance during the core growth phase. μ_{max} was derived from the slope of the linear regression of $\ln(X)$ plotted against time t. The time points used in the regression are mentioned in Table 1.

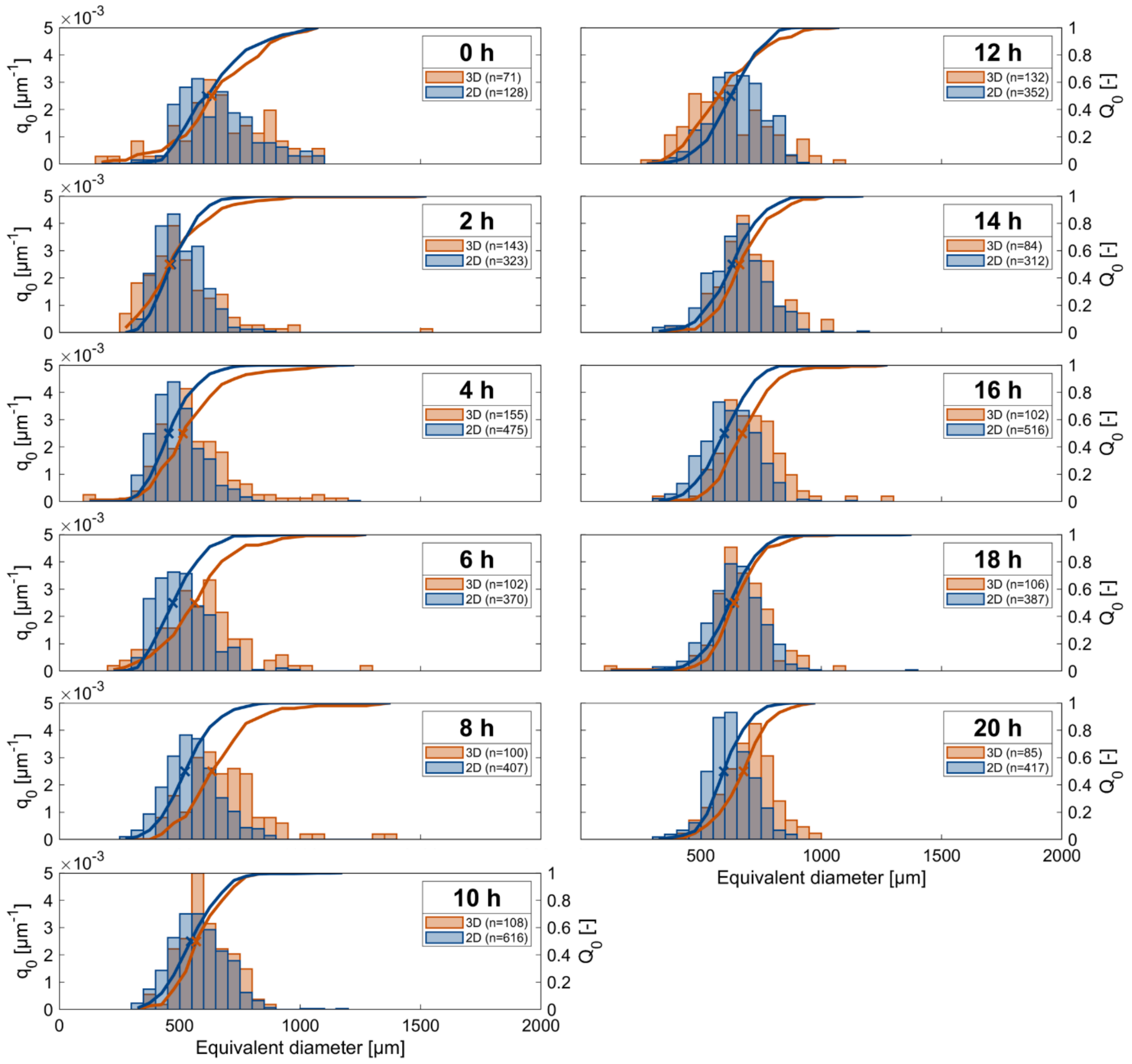

Fig. S7a Comparison of equivalent pellet diameter distributions obtained from 2D (area based) and 3D (volume based) image analysis for the cultivation of STR 1 g L^{-1} (A). Histograms represent normalized frequency distributions (q_0) with a bin size of 50 µm, while the solid lines show the corresponding cumulative distributions (Q_0). The '"X" indicates the median diameter and (*n*) indicates the number of analysed pellets for each method.

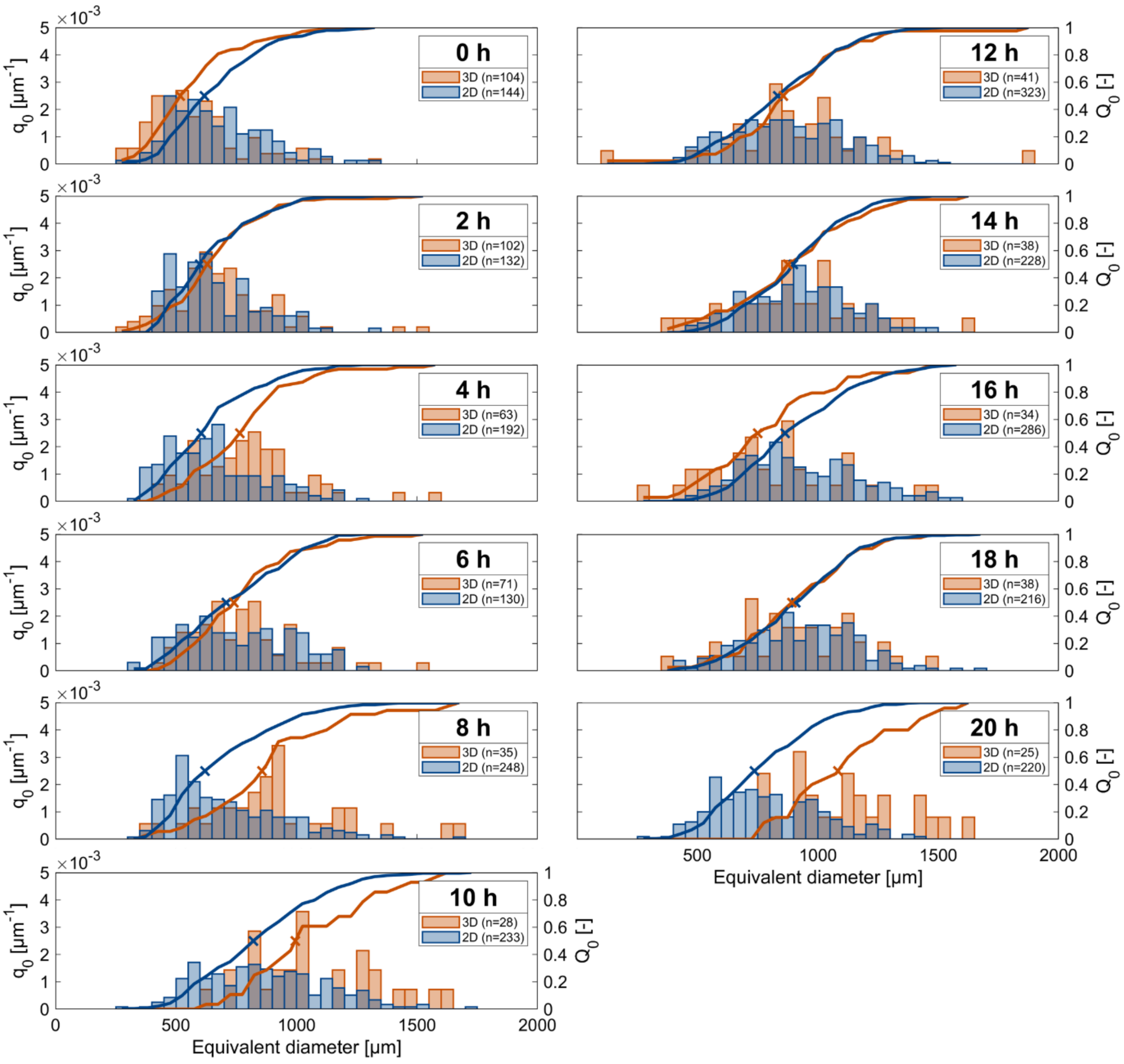

Fig. S7b Comparison of equivalent pellet diameter distributions obtained from 2D (area based) and 3D (volume based) image analysis for the cultivation of RMB 1 g L^{-1} (A). Histograms represent normalized frequency distributions (q_0) with a bin size of 50 µm, while the solid lines show the corresponding cumulative distributions (Q_0). The "X" indicates the median diameter and (*n*) indicates the number of analysed pellets for each method.

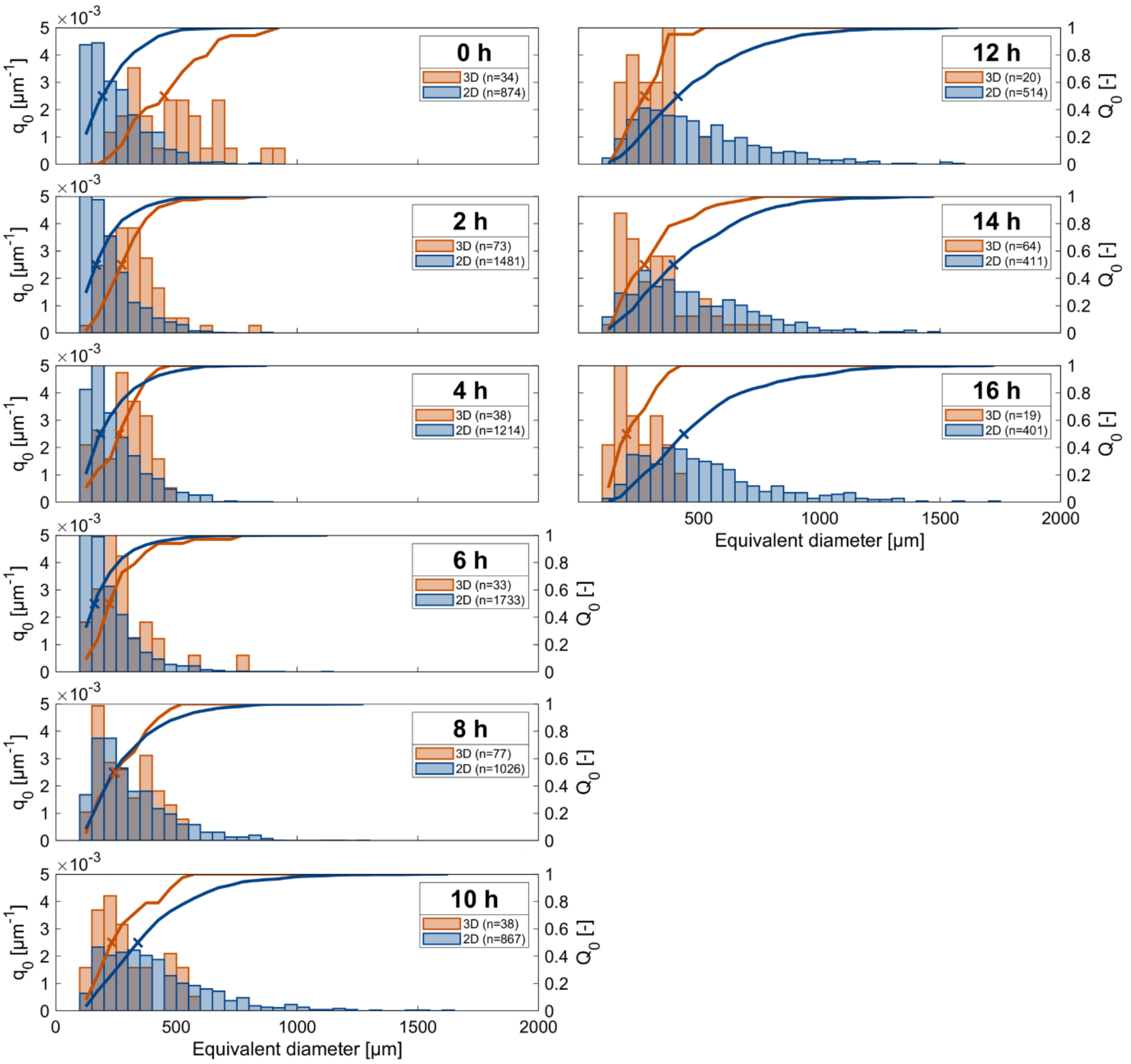

Fig. 7c Comparison of equivalent pellet diameter distributions obtained from 2D (area based) and 3D (volume based) image analysis for the cultivation of STR 10 g L^{-1} (A). Histograms represent normalized frequency distributions (q_0) with a bin size of 50 µm, while the solid lines show the corresponding cumulative distributions (Q_0). The "X" indicates the median diameter and (*n*) indicates the number of analysed pellets for each method. For 18 and 20 h no 3D data are available.

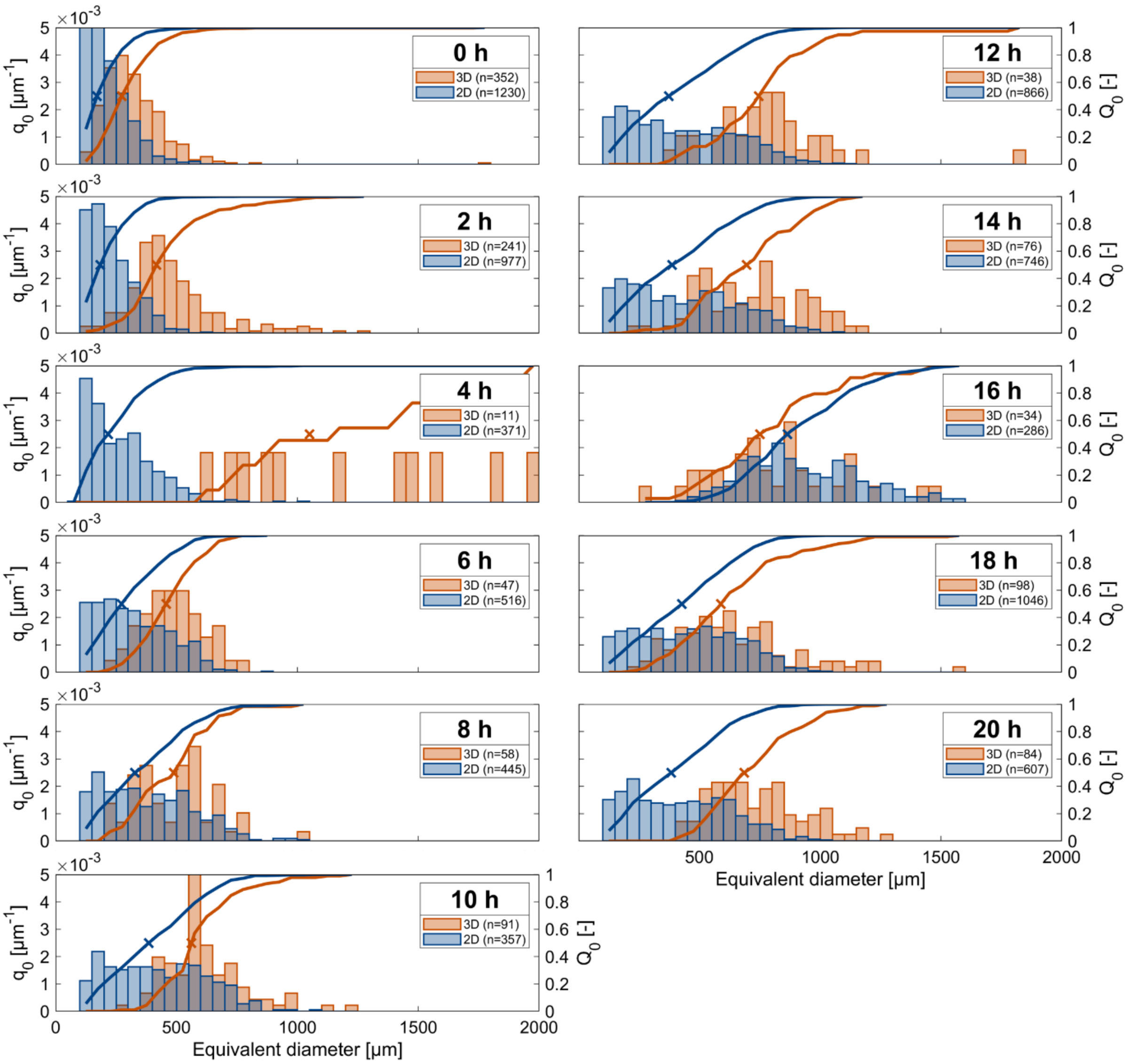

Fig. S7d Comparison of equivalent pellet diameter distributions obtained from 2D (area based) and 3D (volume based) image analysis for the cultivation of RMB 10 g L^{-1} (A). Histograms represent normalized frequency distributions (q_0) with a bin size of 50 µm, while the solid lines show the corresponding cumulative distributions (Q_0). The "X" indicates the median diameter and (*n*) indicates the number of analysed pellets for each method. The 3D data at 4 hours of the cultivation time were excluded from the subsequent analysis.

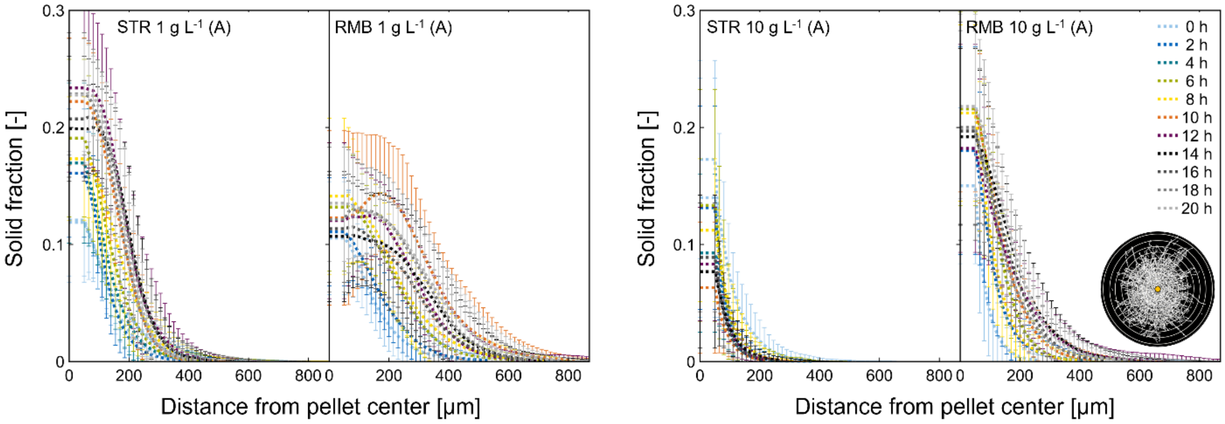

Fig. S8 Given is the mean solid fraction over the pellet radius, as shown in Fig. 6A. In this plot, the standard deviation of each spherical shell was added. For better understanding, shells are visually indicated with an example pellet (black background).

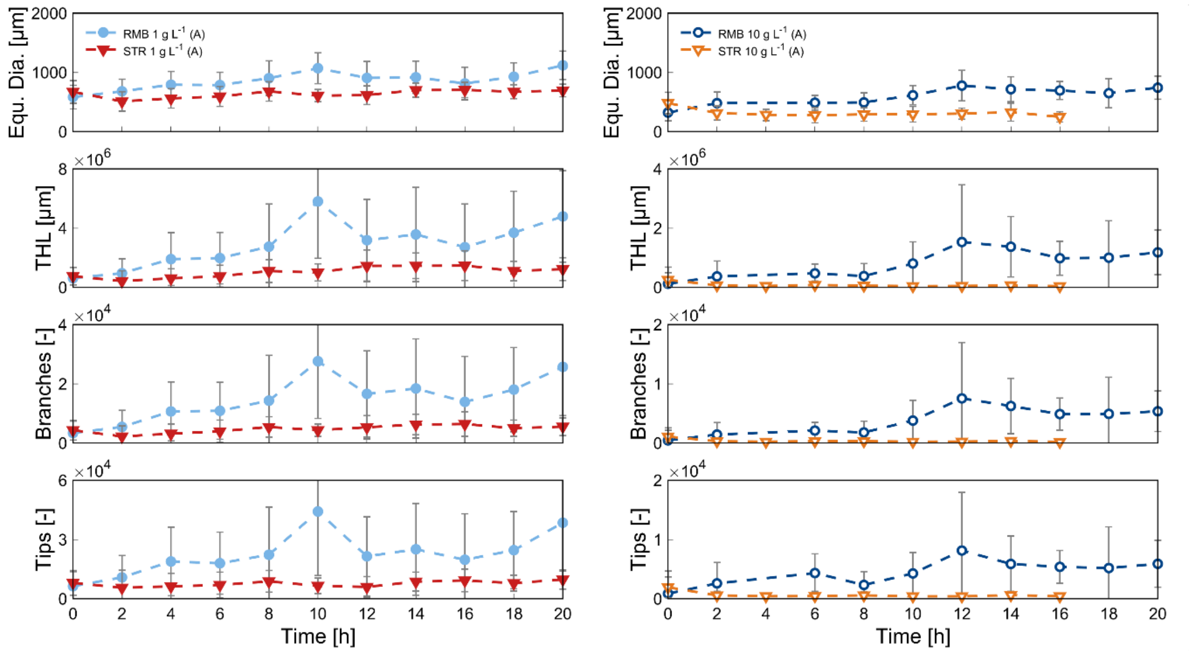

Fig. S9 Mean values and standard deviation of volume based equivalent pellet diameter, total hyphal length (THL), total number of branches, and number of tips per pellet are given over 20 h batch cultures for respective cultivations of replicate A. The dashed lines serve to improve readability.

Reference

Engelbert K, Deffur C, Cairns TC, Zhang F, Kheirkhah T, Winter H, Junne S, Neubauer P, Briesen H, Meyer V (2025) Adjusting *Aspergillus niger* pellet diameter, population heterogeneity, and core architecture during shake flask cultivation. Biotechnol Biofuels Bioprod 18:2661. https://doi.org/10.1186/s13068-025-02661-2
